# Mapping Phylogenetic Trees to Reveal Distinct Patterns of Evolution

**DOI:** 10.1093/molbev/msw124

**Published:** 2016-06-24

**Authors:** Michelle Kendall, Caroline Colijn

**Affiliations:** ^1^Department of Mathematics, Imperial College London, London, United Kingdom

## Abstract

Evolutionary relationships are frequently described by phylogenetic trees, but a central barrier in many fields is the difficulty of interpreting data containing conflicting phylogenetic signals. We present a metric-based method for comparing trees which extracts distinct alternative evolutionary relationships embedded in data. We demonstrate detection and resolution of phylogenetic uncertainty in a recent study of anole lizards, leading to alternate hypotheses about their evolutionary relationships. We use our approach to compare trees derived from different genes of Ebolavirus and find that the VP30 gene has a distinct phylogenetic signature composed of three alternatives that differ in the deep branching structure.

*Key words:* phylogenetics, evolution, tree metrics, genetics, sequencing.

## Introduction

A fundamental challenge in the study of evolution is that for a given set of organisms, markedly different phylogenetic trees can be inferred from each combination of input data, software, and settings (Sullivan et al. 1996; Rokas et al. 2003; Jiang et al. 2014). Reasons for this include lack of informative data, differences between tree inference methods, conflicting signals from descent and selection (convergent evolution), and the fact that evolution is not always tree-like: Gene trees differ from species trees, and many organisms exchange genes horizontally. Phylogenetic uncertainty is often apparent following Bayesian Markov Chain Monte Carlo (MCMC) inference of trees from data (e.g., BEAST [Drummond et al. 2012] and MrBayes [Huelsenbeck and Ronquist 2001]). These tools produce large posterior collections of trees which can include considerable variety and are therefore hard to summarize. Phylogenetic incongruence describes the (often related) issue of conflicting trees from different loci, for example, individual gene trees carrying different evolutionary signals.

Direct qualitative comparison of trees is illustrative but becomes unwieldy and uninformative when the trees are large or differ considerably. Approaches to direct comparison include visualizing the differences between two trees with tanglegrams and exploring the differences in collections of trees using consensus networks (Holland et al. 2005) (for unrooted trees) or DensiTree (Bouckaert 2010) plots (for rooted trees). These visualizations can be challenging to interpret when there are large numbers of trees to be compared. Quantitative, metric-based tree comparisons are an alternative to visual methods, but they currently suffer from drawbacks including counterintuitive behavior (Kuhner and Yamato 2014) and poor resolution (Hillis et al. 2005). For example, in the widely used Robinson–Foulds (RF) unweighted metric (Robinson and Foulds 1981), also known as the “symmetric difference,” many pairs of trees are the same distance apart, and large distances between trees do not imply large differences among the shared ancestry of most tips (Steel and Penny 1993). These limitations hamper the examination of Bayesian posterior collections of trees, so posterior distributions are typically summarized with a single maximum clade credibility (MCC) tree together with edge support values that describe the location and extent of uncertainty. How that uncertainty arises from the ancestral patterns in the data is not revealed; using a single summary tree carries the drawback that crucial information can be lost (Heled and Bouckaert 2013).

A “metric” is a mathematical notion of distance; specifying a metric gives structure and shape to a set of objects. Each metric on a set of trees defines a “tree space.” The size and complexity of tree spaces present serious challenges; there are (2k−3)!! possible topologies for rooted, binary trees with *k* labeled tips (Rohlf 1983). As an illustration, this means that there are 34,459,425 trees with just 10 tips, and 10^76^ trees with 50 tips.

Here we present an approach to compare and cluster groups of trees. Central to our approach is a tree metric which lends itself to clear visualizations of tree space in low dimensions. It enables straightforward detection of distinct groups of similar trees. Accordingly, our method provides a natural solution to the problem of summarizing complex tree spaces, producing a small number of representative trees that capture distinct patterns of evolution reflected in the data.

## New Approaches

**Fig. 1.**
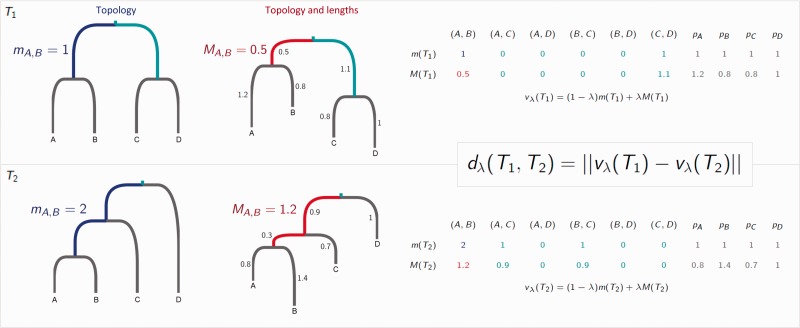
A tree is characterized by the vectors *m* and *M*, which are calculated as shown. These are used to calculate the distance between two trees for any λ∈[0,1]. Here, $d_{0}(T_{1},T_{2})=2$ and $d_{1}(T_{1},T_{2})=1.96$.

Our metric compares the placement of the most recent common ancestor (MRCA) of each pair of tips in two trees. The trees must be rooted and have identical sets of tips labels. We will use *k* to denote the number of tips in each tree. We record the distance between the MRCA of a pair of tips (*i*, *j*) and the root, in each tree, in two ways: The number of edges $m_{i,j}$, and the path length $M_{i,j}$ (fig. 1). We also record the length *p_i_* of each “pendant” edge between a tip *i* and its immediate ancestor. This procedure results in two vectors for a tree *T*:

$$\begin{array}{l} m(T)=(m_{1,2},m_{1,3},\ldots ,m_{k-1,k},\underset{k\text{times}}{\underset{︸}{1,\ldots ,1}}),\\ M(T)=(M_{1,2},M_{1,3},\ldots ,M_{k-1,k},p_{1},\ldots ,p_{k})\;. \end{array}$$

In *m*(*T*) we have recorded each pendant length as 1, as each tip is 1 step from its immediate ancestor. The vector *M*, which is similar to the vector of cophenetic values (Sokal and Rohlf 1962; Cardona et al. 2013) (see supplementary material, Supplementary Material online) depends on the tree’s branch lengths, whereas *m* only depends on its structure or “topology.” We combine *m* and *M* with a parameter λ∈[0,1], which determines how much the topology of the tree only (*λ* = 0), versus the tree with branch lengths (*λ* = 1), contributes. In this way, we capture each tree with a vector $v_{\lambda }(T)=(1-\lambda )m(T)+\lambda M(T)$. The distance between two trees—our metric function—is the Euclidean distance between these vectors:

$$d_{\lambda }(T_{a},T_{b})=||v_{\lambda }(T_{a})-v_{\lambda }(T_{b})||.\;$$

A more formal description and proof that this is a metric are given in the supplementary material, Supplementary Material online.

There are various techniques available to use distances between a set of objects (in this case, trees) to visualize, group, and compare them. For example, multidimensional scaling (MDS) (Cox TF and Cox MAA 2000) is a technique that projects distances in such a way as to best capture them with just a few (e.g., two or three) dimensions. Given a reliable projection we can visually explore comparisons between trees (Amenta and Klingner 2002; Hillis et al. 2005; Holmes 2006; Berglund 2011; Chakerian and Holmes 2012), for example, using color to differentiate trees derived from different genes or data sources, and assessing whether or not they form distinct groups in the space. Alternatively, within a collection of trees such as a Bayesian posterior, clustering algorithms such as *k*-means or nearest-neighbor clustering can use the distances to detect groups of similar objects (so that within-group distances are smaller than between-group distances). These tools do not rely on projections; there is an extensive literature on methods for robust clustering and cluster significance (Legendre P and Legendre LFJ 2012). Here, we use MDS projection to illustrate our metric’s distances between trees. We use *k*-means clustering applied to the distances, and choose the number of clusters so as to optimize the Bayesian Information Criterion (BIC). We illustrate our approach using three data sets: The set of all possible trees with six tips, species trees of anole lizards (Geneva et al. 2015a, 2015b), and trees of Ebolavirus sequences (Gire et al. 2014).

The metric also provides a convenient way to select a representative summary tree (or a summary tree per cluster) using the geometric median tree. Unlike other summary tree methods, this produces representative tree(s) selected from the original tree set with well-supported, nonnegative branch lengths.

In the supplementary material, Supplementary Material online, we prove that the distance we define is a metric and discuss further its properties and possible extensions (supplementary section S1), relate it to other approaches to tree comparisons (supplementary section S2), and provide supplementary analyses and results (supplementary section S3).

## Results

**Fig. 2.**
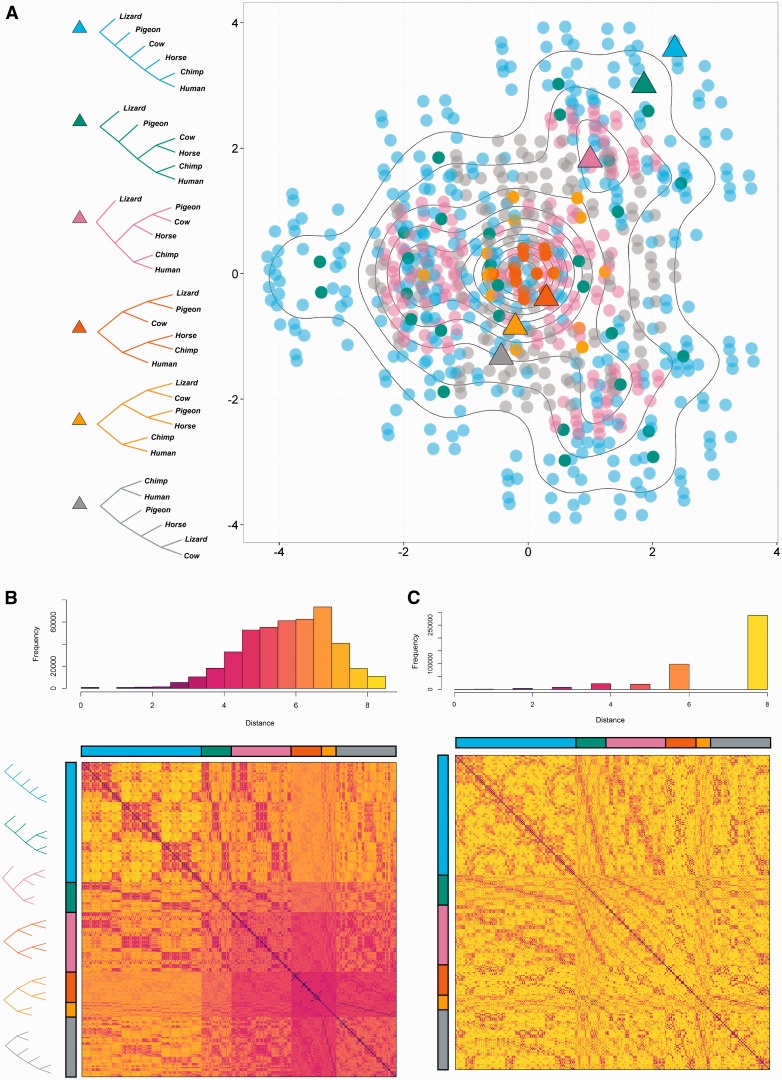
A comparison of all 945 trees on six tips using our metric (*λ*  = 0). (*A*) MDS visualization of tree distances when *λ*  = 0. Colors correspond to tree shapes, of which examples are shown with triangles. Symmetries correspond to permutations of the labels. As the projection often requires that multiple trees are plotted at the same co-ordinates, contour lines are used to indicate density of points. (*B*) Distance matrix for these trees (*λ* = 0), with trees grouped according to shape as indicated by the color bars. (*C*) Unweighted RF distance matrix for the same trees. Most (64%) of the tree pairs are at RF distance 8.

Figure 2*A* gives a visualization using MDS of the topological (*λ*  = 0) distances in our metric between all 945 phylogenetic trees on six tips. In the plot each point represents a tree, and the distance between any pair of points approximates the distance between them given by the metric when *λ*  = 0. Our metric captures differences in both shape and labeling, and preserves symmetries of distances between label permutations. It produces a wide range of tree distances and captures intuitive similarities (e.g., the similar chimp–human pairing in the yellow and gray triangles in figure 2*A*, basal to the other species). In figure 2*C* we compare our tree distances with those of the unweighted RF metric because this is currently the most widely used topological metric (branch lengths are not considered, so it is directly comparable to our metric with *λ*  = 0). However, it is designed for unrooted trees, whereas our approach captures the root placement. In supplementary section S2 and figures S3–S7, Supplementary Material online, we provide comparisons to other tree metrics, including those which take into account branch lengths and/or root position.

### Anole Lizards

**Fig. 3.**
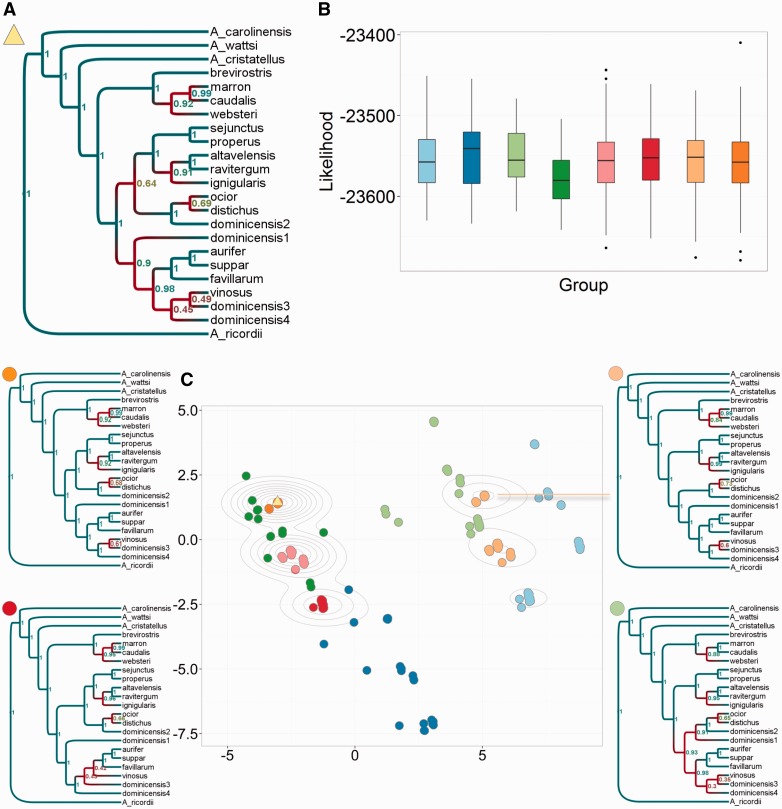
Identifying and exploring islands in anoles *BEAST trees. (*A*) MCC tree for the whole posterior. (*B*) Boxplots of the *BEAST log-likelihood scores of the sampled trees, separated and colored by cluster. The log-likelihood distributions were comparable for each cluster. (*C*) MDS plot of 1,000 trees from the posterior, colored according to eight clusters found using *k*-means clustering. Four examples of an MCC tree for an individual cluster are given here. A more visually dispersed cluster corresponds to more uncertainty in larger clades. The relatively small number of points in the plot (≪1,000) corresponds to the small number of distinct topologies explored; density of points is illustrated with contour lines.

In the evolution and ecology of higher organisms, phylogenetic trees are used to uncover the origins and adaptations of existing species. This is greatly hindered by the difficulty in resolving species trees using nuclear DNA from different loci and/or mitochondrial DNA. Loci may not contain sufficient variation to estimate fully resolved trees, and often result in discordant trees. Anole lizards in particular are a model system for ecological phenomena including reproductive character displacement, adaptation, behavior, and speciation (Geneva et al. 2015b). Recently, Geneva et al. performed the first comprehensive phylogenetic analysis of the *distichus* group of trunk ecomorph anoles (based on mitochondrial and nuclear DNA) (Geneva et al. 2015a, 2015b). They tested three key hypotheses concerning the biogeographical origins and boundaries of anoles from Hispaniola and the Bahamas, and the evolution of their dewlap coloration. There were two main areas of uncertainty in the species tree, reflected in clades in the MCC with low support values (fig. 3*A*).

We computed pairwise tree distances according to our metric (*λ*  = 0) for a random sample of 1,000 posterior species trees from a *BEAST analysis (fig. 3*C*). It is immediately apparent from a 2D MDS visualization that the posterior is not unimodal, but contains distinct clusters of tree topologies. Using *k*-means clustering, we identified eight distinct groups of trees, most of which are arranged in visually separated clusters, particularly when shown in 3D (supplementary fig. S9, Supplementary Material online). The MCC tree (fig. 3*A*) is situated in the center of the largest cluster. There are many trees with the same topology as the MCC tree, so it appears in figure 3*C* as one point surrounded by multiple contour lines, to illustrate the density of points. As the clusters of trees have comparable posterior probabilities (fig. 3*B*), there is no reason (in the absence of further research) to exclude the alternative clusters to the MCC tree, which alone cannot capture the distinct, likely patterns of evolution supported by the data.

We therefore computed an individual MCC tree for each cluster, to illustrate the clusters’ alternative arrangements of the taxa. The support values for clades in cluster-specific MCC trees are high. In other words, the clusters correspond to alternative resolutions of uncertain clades in the posterior. This result is not limited to the anole data set; we also find clusters corresponding to distinct, likely evolutionary histories for a variety of data sets including species trees of chorus frogs (see supplementary material, Supplementary Material online). We find that where a cluster is tightly defined in the metric, the corresponding support values are higher than they are if the cluster is loosely defined (supplementary fig. S11, Supplementary Material online).

The key differences between the anole tree clusters are in the placement of the *ocior*, *distichus*, *dominicensis2* clade, which in the MCC tree and on the left-hand side of figure 3C is sister to the *sejunctus*–*ignigularis* clade, but on the right-hand side is sister to the *dominicensis1*–*dominicensis4* clade. These alternative placements affect the likely origins of the Bahamian anoles *ocior* and *distichus*, placing them closer in their evolutionary history to the anoles of the North or South Paleo-island of Hispaniola, respectively (see supplementary material, Supplementary Material online). The placement of *dominicensis1* and the topology of the *aurifer*–*dominicensis4* clade also vary between clusters. For each of the eight representative trees, we retested the hypothesis that evolution of dewlap color between pale yellow and dark red has occurred repeatedly across the species group. All eight trees supported this hypothesis, but the inferred transition rates and ancestral coloring of major clades differed between the clusters (see supplementary material, Supplementary Material online).

We compared the analysis derived from our metric to a similar pipeline using the RF metric and found that it does not resolve the posterior into a manageable number of distinct, well-supported patterns of evolution (see supplementary material, Supplementary Material online). In addition, the MDS-projected distances of the RF metric are not very well correlated to the RF distances. MDS visualizations of RF and Billera, Holmes, Vogtmann (BHV) (Billera et al. 2001) distances between trees were discussed in Holmes (2006), Chakerian and Holmes (2012), Amenta and Klingner (2002), Hillis et al. (2005), and Berglund (2011), but these approaches have been hampered by the limitations of the underlying metrics, as discussed earlier and in the supplementary material, Supplementary Material online.

## Ebolavirus

**Fig. 4.**
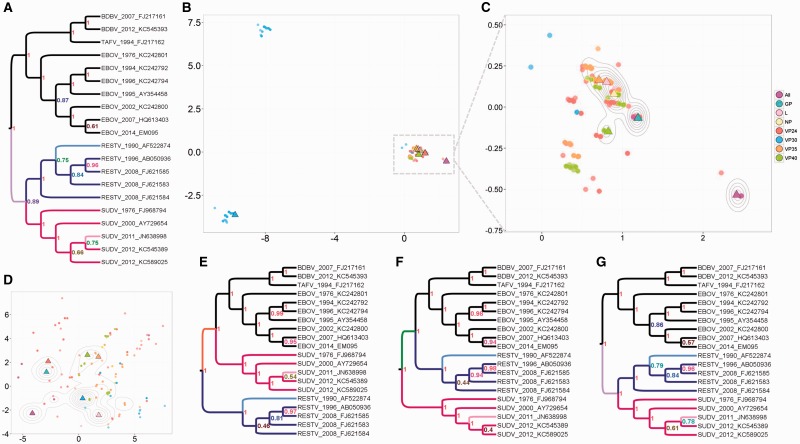
Ebolavirus comparison of individual and “all” gene trees. (*A*) MCC tree from “all” (purple triangle). (*B*) MDS plot of 1,200 trees (150 per type), showing three distinct groups of topologies for VP30. (*C*) A closer look at the largest group from B. The MCC tree per gene is marked as a triangle. The MCC trees for GP and VP24 are plotted in almost the same position, in the center of the largest group among individual gene trees. The distance between them is $\sqrt{2}$, the minimum distance by our metric. (*D*) MDS plot of all 1,200 trees using the RF metric. Distinct VP30 topologies are not detected, in fact, the VP30 MCC tree is identical to the NP MCC tree according to RF because it is an unrooted metric. (*E–G*) MCC tree from lower left VP30 cluster, upper left VP30 cluster, and main cluster, respectively. The MCC tree from the largest cluster, G, is naturally more similar to A than to E or F.

Comparing trees estimated from different genes or loci can play a role in quantifying phylogenetic incongruence and detecting horizontal movement of genes and convergent evolution. We compared trees from the seven genes of Ebolavirus which causes the fatal Ebola hemorrhagic fever. We selected 20 published sequences (Gire et al. 2014) which differed on every gene (including all five viral species of the Ebolavirus genus) and inferred trees in BEAST from each gene separately and from all genes together. Our method shows (fig. 4) that the VP30 gene, an essential viral transcription activator, has a distinctive phylogenetic pattern, forming three distinct clusters with comparable likelihoods (fig. 4*B*). Each cluster of VP30 trees resolves the phylogenetic uncertainty in the placement of the Sudan clade (pink), placing it in one of three positions: Sister to the EBOV, TAFV and BDBV clade, sister to a larger clade containing all the other sequences, or sister to the Reston clade. The latter placement, which is also found in all the MCC trees other than VP30, agrees with previous analysis (Dudas and Rambaut 2014; Gire et al. 2014). Each cluster places the Reston and Sudan sequences into monophyletic clades, but there are differences in the placement of the Reston 1990 and Sudan 2011 tips.

The alternative phylogenetic signals from VP30, with differences in the deep structure of the tree, suggest that there is something distinct in the deep ancestry among these sequences in the VP30 gene that is not shared with the other genes (which in turn are highly congruent with each other). Our method simply detects this distinctive phylogenetic signal in a group of trees. The signal may be the result of a historical recombination event, convergent evolution, or lack of sufficient information in this gene to reconstruct the deep ancestry of the taxa (though if that were the case we would not expect to have found such tight and distinctive clusters, and posterior probabilities comparable to the other genes). Although an extensive comparison of tools to detect recombination is outside the scope of this work, we used the standard tool GARD (Pond et al. 2006), which compares neighbor-joining trees, and it did not detect recombination among these sequences.

Marzi et al. recently found that a new whole-cell vaccine, EBOVΔVP30, is safe and effective against lethal Ebola challenge in nonhuman primates (Halfmann et al. 2008; Marzi et al. 2015). They also did not observe recombination in relatively short-term experiments (based on viral passage). However, if VP30 is amenable even to rare recombination events or rapid convergent evolution in this genus, this could threaten the future efficacy of EBOVΔVP30 by allowing Ebolavirus to generate vaccine escape strains. Accordingly, it is important to have tools which can rapidly uncover phylogenetic incongruence and analyze phylogenetic uncertainty.

## Discussion

Our method reveals distinct patterns of evolution, both in viruses and in higher organisms. It exposes the extent to which choosing a single summary tree discards other well-supported alternatives. It allows quantitative comparisons between the evolutionary patterns of different genes or loci. Further, it can measure the extent to which a particular locus shapes the most likely phylogenetic trees for a set of data, and thereby identify phylogenetically informative sites, loci or genes. It provides a framework for comparing and testing tree estimation methods, particularly where these produce rooted trees or where an outgroup is available (see supplementary results, Supplementary Material online, for dengue virus). Addressing tree estimation challenges is increasingly important as data sets grow to tens of thousands of tips, rendering standard inference methods infeasible. More generally, our method is relevant to any rooted, labeled trees with the same set of tips, including decision trees, network spanning trees, hierarchical clustering trees, and language trees. The method expands the toolkit for tree comparisons. It is implemented within the R package “treescape” (Jombart et al. 2015), which also makes other tree metrics available for use in the same pipeline.

The example in figure 2 of the space of trees with six tips demonstrates that, while the space is not continuous, vectors which correspond to trees are densely packed together. This helps to show that when we do see “gaps” between clusters of trees, for example in figure 3, these truly correspond to parts of tree space which have either been rejected or not explored by the inference process. The Ebolavirus example in figure 4 demonstrates that some Bayesian posteriors do center around a single tree topology without widely separated clusters. This is seen most strongly for the trees from all genes together, which vary only marginally, and is true for most of the individual genes. The appearance of widely separated clusters in other trees, such as those from the VP30 gene, is therefore significant and an important consideration for further analysis.

While requiring trees to have a root is in some sense a limitation, many evolutionary analyses are implicitly or explicitly performed in the context of a root. This applies to any analysis with a temporal component, as this defines a direction back through time in the tree, in which the root is the earliest point. Time plays a role in analyses of speciation and diversification rates, species dating analyses, phylodynamic analyses (Stadler et al. 2012; Didelot et al. 2014; Rasmussen et al. 2014), and many others. In addition, in many analyses it is both desirable and straightforward to include an outgroup consisting of taxa known to be phylogenetically distinct from taxa in the data set. This defines a natural root for the data set taxa. In all of these cases, taking the root placement into account in the analysis (and in tree comparisons) is important, and our results suggest that it can substantially improve the resolution and utility of tree comparisons.

In our metric, differences in a tree near the root cause larger tree distances than differences near the tips. Branching events closer to the root are also typically harder to infer than those nearer to the tips. Accordingly, the fact that the metric is sensitive to differences in the deep branching structure allows it to easily detect viable alternative deep tree structures in a collection of trees, and to group the credible alternatives together with their downstream consequences. The height of the root may also be challenging to estimate; different genes may produce trees that are structurally concordant but that differ in the root height. Length differences can easily dominate tree distances in such a case; the flexibility of our metric to explore this by weighting structure versus length allows us to detect this phenomenon (see supplementary material, Supplementary Material online).

Although there is no a priori best way to define a distance between two trees, the MRCAs of tip pairs and clades are of central interest to evolutionary biologists. In our metric, trees that largely share this ancestry are close together. Indeed, in the absence of an underlying “true” distance on the set of trees, a good measure of the quality of a metric must be whether it captures intuitive similarities and has useful applications. The RF distance captures the intuitive property of the number of nearest-neighbor interchanges required to convert one tree into another, but suffers from the fact that in some cases, a single regrafting event can result in a high distance. It also suffers from a lack of resolution (many trees are similar distances apart), limiting its power in many settings. The BHV metric has the significant advantages of convexity and geodesic distances, but has some similar drawbacks to the RF approach. The path difference metric (Steel and Penny, 1993) achieves better resolution of distances but the natural symmetries of the space are not clear from the 2D projection of trees with six tips (supplementary fig. S4, Supplementary Material online). There is little correlation between any of these metrics (supplementary figs. S6 and S7, Supplementary Material online). We compare existing tree comparisons in more detail in the supplementary material, Supplementary Material online. As any positive linear combination of metrics is also a metric, our metric could be used to construct tree distances that flexibly weight a variety of tree characteristics, such as the extent to which the root placement is important (e.g., $d(T_{a},T_{b})=w_{\text{no root}}d_{\text{RF}}(T_{a},T_{b})+w_{\text{root}}d_{\lambda }(T_{a},T_{b})$, with $w_{\text{no root}},w_{\text{root}}>0$).

Sequencing technologies continue to decrease in cost, with the result that it is now feasible to sequence up to tens of thousands of taxa in multiple genes, at least in viruses. As the computational challenges associated with haplotype phasing are resolved (Pan et al. 2014; Zhi and Zhang 2014; Regan et al. 2015), phylogenetic methods will be used for large data sets on higher organisms. As an example, the Epidemiology Network Ag1000G has 765 Anopheles mosquito genomes visible to the public (MalariaGEN 2015). Currently, Bayesian tree inference is not feasible beyond hundreds of taxa, and maximum-likelihood, maximum-parsimony, and neighbor-joining methods are often used with add-on estimation of the root and timing information by software such as Path-O-Gen (Rambaut 2009) and LSD (To et al. 2016). Although this presents challenges for tree inference, it also provides compelling motivation to develop appropriate tree comparison metrics.

The examples we have presented here involve small trees which can be easily viewed. They help to demonstrate how our intuitive understanding of tree differences matches the results of the metric, and to show the relationship between MCC trees, tight clustering, and summary trees. However, the metric can also be applied to trees with thousands of tips. Computing the vector per tree is fast—it is at worst quadratic in the number of tips, when the tree is completely balanced. Of course, computing pairwise distances between a set of *n* trees requires $\left(\begin{array}{c} n\\ 2 \end{array}\right)$ calculations for any metric. For trees with many thousands of tips, the vectors can become infeasibly long and may need to be represented in a more efficient format (as many pairs of tips have the same *m* and *M* values). Exploiting different ways to navigate the tree or to reduce the number of pairwise comparisons made may also yield more efficient computation.

Obtaining credible trees that capture the relationships present in complex data is one of the fundamental open challenges in evolution today (Heled and Bouckaert 2013), as markedly different trees with conflicting evolutionary messages can result from a single data set (Sullivan et al. 1996; Rokas et al. 2003; Jiang et al. 2014). Our metric adds to the toolkit available to solve this problem, capturing distinct evolutionary stories embedded in data and comparing corresponding trees in a quantitative way.

## Materials and Methods

### Anole Lizards

We used species trees from a recent *BEAST analysis of the *distichus* species group in the lizard genus *Anolis*. Geneva et al. (2015b) made species trees available (Geneva et al. 2015a). They sampled 54 individuals from the *brevirostris* (8) and *distichus* (46) complexes, both within the *distichus* species group. For each individual, they sequenced DNA from seven exonic nuclear loci and from one mitochondrial locus. They used gene trees to identify putative species and generated species trees in *BEAST (Heled and Drummond 2010) using four independent analyses, each with 2 billion generations.

We sampled 1,000 trees uniformly at random from the latter half of the available *BEAST posterior (files Anoles_StarBEAST_posterior.species.trees and Anoles_StarBEAST_MCC.species.tre for the posterior trees and MCC tree, respectively, from Geneva et al. [2015a]). We computed all pairwise tree distances according to our metric (*λ*  = 0) in this sample of 1,000 posterior trees. To detect clusters we used *k*-means clustering (using the function find.clusters from adegenet, which calls kmeans from the *stats* package in R [R Development Core Team 2008]), and compared clustering solutions with the BIC, as described in the *adegenet* package in R (Jombart 2008). We found that a choice of *k*  = 8 clusters minimized the BIC. We visualized the distances using MDS (dudi.pco in the *ade4* package in Chessel et al. [2004]). Each point represents a tree, and the distances between the points approximate the distances in our metric. We colored points according to their *k*-means cluster. An MCC tree was found for the whole posterior and for each cluster using TreeAnnotator (Drummond and Rambaut 2007) and plotted with FigTree (Rambaut 2006).

We also tested a variety of other clustering methods including ward.D, ward.D2, single, complete, UPGMA, WPGMA, and WPGMC from hclust in the *stats* package in R, where more details on each of these methods can be found in the documentation. The clustering of the trees naturally varies slightly between methods. However, the important conclusions are insensitive to the clustering method: 1) The tighter the cluster, the more similar the trees (with any variation nearer the tips) and 2) there are about six tight clusters, corresponding to the regions in figure 3 within contour lines. Note that the contour lines are a result of the MDS projection and are therefore independent of the clustering method.

Different clustering methods in tree space can now be tested easily using the function findGroves in the package *treescape* (Jombart et al. 2015), which was developed after we performed this analysis.

## Ebolavirus

We analyzed sequence data from Ebolavirus samples, both historical and from the 2014 outbreak, published recently by Gire et al. (2014). A full description of the data collection, library construction, sequencing, single nucleotide polymorphism calling, and alignments is available in that work (with sequence data and Beast inference settings in file 2014_GN.SL_SRD.HKY_strict_ctmc.exp.xml). We selected the following 20 taxa:

BDBV_2007_FJ217161,  BDBV_2012_KC545393,

EBOV_1976_KC242801, EBOV_1994_KC242792,

EBOV_1995_AY354458, EBOV_1996_KC242794,

EBOV_2002_KC242800, EBOV_2007_HQ613403,

EBOV_2014_EM095,    RESTV_1990_AF522874,

RESTV_1996_AB050936, RESTV_2008_FJ621583,

RESTV_2008_FJ621584, RESTV_2008_FJ621585,

SUDV_1976_FJ968794, SUDV_2000_AY729654,

SUDV_2011_JN638998, SUDV_2012_KC545389,

SUDV_2012_KC589025, TAFV_1994_FJ217162.

These had no duplicated sequences for any gene among the other selected taxa, allowing inference of trees from each gene separately. Following Gire et al. (2014), we used a coalescent prior with exponential growth, a random starting tree, a strict molecular clock with uniform rate across branches and prior mean of 0.0001. We used an HKY (Hasegawa–Kishino–Yano) substitution model with equal rates. We ran 10 million MCMC iterations and confirmed the results with multiple runs in BEAST v1.8. We used a partitioned Beast analysis to infer trees from all genes together (fixing the trees to be shared across partitions).

We sampled 150 trees from the posterior for each gene (1,200 trees in total; fig. 4 in the main text). We computed pairwise distances (*λ*  = 0) between all these trees and visualized them with MDS, coloring the points according to the gene giving rise to the corresponding tree. The results (the three clusters of trees from VP30, and congruence of the other trees) do not depend on the random sampling of 150 trees from the posterior.

## Supplementary Material

Supplementary material, table S1, and figures S1–S16 are available at *Molecular Biology and Evolution* online (http://www.mbe.oxfordjournals.org/).

Supplementary Data
