# Supplementary material for: Mapping Phylogenetic Trees to Reveal Distinct Patterns of Evolution
Source: Mol Biol Evol. 2016 Jun 24;33(10):2735–43. doi: 10.1093/molbev/msw124 (PMC5026250; doi:10.1093/molbev/msw124)
Supplement: Supplementary Data [file supp_33_10_2735__index.html]

Mapping Phylogenetic Trees to Reveal Distinct Patterns of Evolution — Mapping Phylogenetic Trees to Reveal Distinct Patterns of Evolution — Supplementary Data 

# Mapping Phylogenetic Trees to Reveal Distinct Patterns of Evolution

## Supplementary Data

files

- Supplementary Data - pdf file
