# Supplementary material for: Mapping Phylogenetic Trees to Reveal Distinct Patterns of Evolution
Source: Mol Biol Evol. 2016 Jun 24;33(10):2735–43. doi: 10.1093/molbev/msw124 (PMC5026250; doi:10.1093/molbev/msw124)
Supplement: Supplementary Data [file supp_msw124_KendallColijnSupp.pdf]

Supplementary Information

In this supplementary information we provide further details of our method (Section 1): we prove that the distance function we define is a metric; discuss the role of the parameter λ; describe further properties and extensions of our metric; and explain in more detail MDS projections and how to select summary trees. We explain the relationship of our method to existing tree comparison methods in the literature (Section 2). Finally, we provide further results about anole lizards and Ebolavirus, and analysis of additional datasets of chorus frogs and dengue (Section 3).

1 Supplementary details of the method

1.1 Definition of $d_\lambda(T_a, T_b)$ and proof that it is a metric

Let $\mathcal{T}_k$ be the set of all rooted trees on k tips with labels $1, \ldots, k$. Note that we do not require the trees to be binary (bifurcating); they may contain polytomies (multifurcations). In common with previous literature [Harding, 1971, Robinson and Foulds, 1981] we say that trees $T_a, T_b \in \mathcal{T}_k$ have the same labelled shape or *topology* if the set of all tip partitions admitted by internal edges of T_a is identical to that of T_b, and we write this as $T_a \cong T_b$. Equivalently, if we define a tip partition admitted by an internal *node* as the tip partition given by removing an internal node and its incident edges, then $T_a \cong T_b$ if and only if they have the same set of tip partitions admitted by internal nodes. We say that $T_a = T_b$ if they have the same topology and each corresponding branch has the same length.

A function $d : \mathcal{T}_k \times \mathcal{T}_k \to \mathbb{R}$ is a metric if, for all $T_a, T_b \in \mathcal{T}_k$,

1. $d(T_a, T_b) \geq 0$ (distances are non-negative)
2. $d(T_a, T_b) = 0 \Leftrightarrow T_a = T_b$ (the distance is only 0 if they are the same)
3. $d(T_a, T_b) = d(T_b, T_a)$ (distance is symmetric)
4. for any T_c, $d(T_a, T_b) \leq d(T_a, T_c) + d(T_c, T_b)$ (the triangle inequality).

For any tree $T \in \mathcal{T}_k$ let $m_{i,j}$ be the number of edges on the path from the root to the most recent common ancestor (MRCA) of tips i and j, let $M_{i,j}$ be the length of this path, and let p_i be the length of the pendant edge to tip i. Then, including all pairs of tips, we have two vectors:

$$m(T) = (m_{1,2}, m_{1,3}, \ldots, m_{k-1,k}, \underbrace{1, \ldots, 1}_{k \text{ times}}) \ ,$$

which captures the tree topology, and

$$M(T) = (M_{1,2}, M_{1,3}, \ldots, M_{k-1,k}, p_1, \ldots, p_k)$$

which captures the topology and the branch lengths. We form a convex combination of these, parameterised with $\lambda \in [0, 1]$, to give

$$v_\lambda(T) = (1 - \lambda) m(T) + \lambda M(T) \ .$$

Theorem 1. *The function $d_\lambda : \mathcal{T}_k \times \mathcal{T}_k \to \mathbb{R}$ given by*

$$d_\lambda(T_a, T_b) = \| v_\lambda(T_a) - v_\lambda(T_b) \|$$

is a metric on $\mathcal{T}_k$, where $\| \cdot \|$ is the Euclidean distance (l^2-norm) and $\lambda \in [0, 1]$.

Proof of Theorem 1. Since the Euclidean distance between vectors satisfies three of the necessary conditions for being a metric (non-negative, symmetric and obeying the triangle inequality) it remains to prove condition 2. Since the vectors are well-defined it is clear that $T_a = T_b \Rightarrow d_\lambda(T_a, T_b) = 0$. Thus it remains to prove that $d_0(T_a, T_b) = 0 \Rightarrow T_a \cong T_b$ (i.e. the $\lambda = 0$ distance is 0 only when the trees have the

same topology) and $d_\lambda(T_a, T_b) = 0 \Rightarrow T_a = T_b$ for all $\lambda \in (0, 1]$ (i.e. for $0 < \lambda \leqslant 1$ the distance is 0 only when the trees are identical). We will address this in three stages, showing that (1) the tree topology vector, (2) the branch-length focused vector, and (3) their convex combination each uniquely define a tree. That is, we show that for $T_a, T_b \in \mathcal{T}_k$,

1. $m(T_a) = m(T_b) \Rightarrow T_a \cong T_b$,
2. $M(T_a) = M(T_b) \Rightarrow T_a = T_b$, and
3. for $\lambda \in (0, 1), v_\lambda(T_a) = v_\lambda(T_b) \Rightarrow T_a = T_b$.

For ease of notation we restrict our attention here to binary trees; it is straightforward to extend these arguments to trees that are not binary, replacing mention of 'left' and 'right' leaf sets L and R descending from an internal node by a list of descendant leaf sets $S_1, S_2, \ldots, S_p$ for a polytomy of p descendant branches.

1. We show that $m(T)$ characterises a tree topology. Suppose that for $T_a, T_b \in \mathcal{T}_k$ we have $d_0(T_a, T_b) = 0$, so $m_{i,j}(T_a) = m_{i,j}(T_b)$ for all pairs $i, j \in \{1, \ldots, k\}$. Consider the tip partition created by the root of T_a. That is, if the root and its two descendant edges were removed, then T_a would be split into two subtrees, whose tip sets we label L and R. For all leaf pairs (i, j) with $i \in L$ and $j \in R$ we have $m_{i,j}(T_a) = 0$, and therefore $m_{i,j}(T_b) = 0$. Thus the root of T_b also admits the leaf partition $\{L, R\}$.

Similarly, any internal node n in T_a partitions its descendant tips into non-empty sets which we can call L_n, R_n. Let the number of edges on the path from the root to n in T_a be x_n. Notice that for any pair of leaves in T_a where one leaf is in L_n and the other is in R_n, their MRCA is the internal node n. That is, for all leaf pairs (i, j) with $i \in L_n$, $j \in R_n$ we have $m_{i,j}(T_a) = x_n$. Since $m(T_a) = m(T_b)$ for all (i, j) we also have $m_{i,j}(T_b) = x_n$ for the leaf pairs (i, j) with $i \in L_n$, $j \in R_n$. This means that there must be an internal node in T_b which also partitions the leaves into the sets L_n, R_n, at an edge of distance x_n from the root. Since this is true for all internal nodes we have $T_a \cong T_b$, and d_0 is a metric on tree topologies. Note that the final k fixed entries of $m(T)$ are redundant for unique characterisation of the topology of the tree, but are included to allow the convex combination of the topological and branch-length focused vectors.

2. We show that $M(T)$ characterises a tree using a similar argument to that of part (1). Suppose that for $T_a, T_b \in \mathcal{T}_k$ we have $d_1(T_a, T_b) = 0$, so $M_{i,j}(T_a) = M_{i,j}(T_b)$ for all pairs $i, j \in \{1, \ldots, k\}$. Let the *length* of the path from the root to internal node n be X_n. Then for all $i \in L_n$, $j \in R_n$ we have $M_{i,j}(T_a) = X_n = M_{i,j}(T_b)$, which means that T_b also contains an internal node at distance X_n from the root which admits the partition $\{L_n, R_n\}$. Since this holds for all internal nodes including the root (where $X_n = 0$), we have that T_a and T_b have the same topology and *internal* branch lengths.

The final k elements of $M(T)$ correspond to the pendant branch lengths. When $M(T_a) = M(T_b)$ we have that for each $i \in 1, \ldots, k$ the pendant branch length to tip i has length p_i in both T_a and T_b. Thus T_a and T_b have the same topology and branch lengths, hence $T_a = T_b$ and d_1 is a metric.

3. Finally, we need to show that $v_\lambda(T)$ characterises a tree for $\lambda \in (0, 1)$. Suppose that for $T_a, T_b \in \mathcal{T}_k$ and $\lambda \in (0, 1)$ we have $d_\lambda(T_a, T_b) = 0$, so $v_\lambda(T_a) = v_\lambda(T_b)$.

Each vector has length $\binom{k}{2} + k = \frac{k(k+1)}{2}$. It is clear that for the final k entries, that is for $\frac{k(k-1)}{2} < i \leq \frac{k(k+1)}{2}$ we have

$$0 = (1 - \lambda)(1 - 1) + \lambda(M_i(T_a) - M_i(T_b))$$

which implies that $M_i(T_a) = M_i(T_b)$.

We therefore restrict our attention to the first $\binom{k}{2}$ elements of v_λ. Now $d_\lambda(T_a, T_b) = 0$ implies that

$$0 = (1 - \lambda)(m_{i,j}(T_a) - m_{i,j}(T_b)) + \lambda(M_{i,j}(T_a) - M_{i,j}(T_b)) \quad (1)$$

for all $i, j \in \{1, \ldots, k\}$. Although it is possible for Equation 1 to hold for *some* $i, j \in \{1, \ldots, k\}$ when the trees are different, we will show that for any $\lambda \in (0, 1)$, Equation 1 only holds for *all* $i, j \in \{1, \ldots, k\}$ when $T_a = T_b$.

Suppose for a contradiction that we have $T_a \neq T_b$ but $d_\lambda(T_a, T_b) = 0$. First, observe that whenever $m_{i,j}(T_a) = 0$ then $M_{i,j}(T_a) = 0$ also because the MRCA of i and j is the root. For such pairs (i, j), Equation 1 gives $m_{i,j}(T_b) = M_{i,j}(T_b) = 0$, and so $d_\lambda(T_a, T_b) = 0$ implies that T_a and T_b must share the same root partition. Now fix $\lambda \in (0, 1)$ and consider a pair of tips $x, y \in \{1, \ldots, k\}$ where

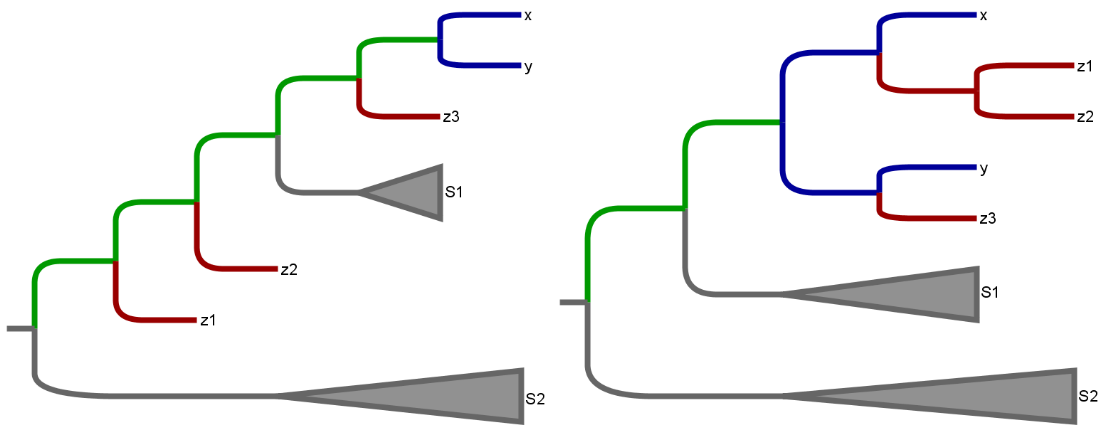

Figure 1: If $d(T_a, T_b) = 0$ then T_a and T_b must share the same root partition, hence S_2 is the same set of tips in both trees. If $m_{x,y}(T_a) \neq m_{x,y}(T_b)$ then $m_{x,y}(T_a) - m_{x,y}(T_b) = n$ for some $n \in \mathbb{N}$. Here $m_{x,y}(T_a) - m_{x,y}(T_b) = 5 - 2 = 3$, so there exist at least 3 tips z_1, z_2, z_3 between the root and the MRCA of x and y in T_a, but positioned further from the root than the MRCA of x and y in T_b.

$m_{x,y}(T_a), m_{x,y}(T_b) \neq 0$ and $m_{x,y}(T_a) \neq m_{x,y}(T_b)$, which must exist since $T_a \neq T_b$, using part 1. Without loss of generality, suppose that $m_{x,y}(T_a) - m_{x,y}(T_b) = n$, where $n \in \mathbb{N}$. Then there exist at least n tips $z_1, \ldots, z_n$ which descend from internal nodes positioned between the root and the MRCA of x and y in T_a, but in T_b they must descend from internal nodes which descend from the MRCA of x and y, as demonstrated in Figure 1. That is, (because the trees have the same root partition) there exist at least n tips $z_1, \ldots, z_n$ such that for each $i \in \{1, \ldots, n\}$

$$m_{x,z_i}(T_a) = m_{y,z_i}(T_a)$$

and

$$m_{x,z_i}(T_a), m_{y,z_i}(T_a) < m_{x,y}(T_a),$$

whilst

$$m_{x,z_i}(T_b) \geq m_{x,y}(T_b), \text{ and } m_{y,z_i}(T_b) \geq m_{x,y}(T_b) \ .$$

Now, pick z_j so that $m_{x,z_j}(T_a) = \min_{i \in [n]} m_{x,z_i}(T_a)$. Then $m_{x,z_j}(T_a) \leq m_{x,y}(T_a) - n$ and so

$$\begin{aligned} m_{x,z_j}(T_a) - m_{x,z_j}(T_b) &\leq (m_{x,y}(T_a) - n) - m_{x,y}(T_b) \\ &= n - n \\ &= 0 \ . \end{aligned}$$

Now since Equation 1 holds for all $i, j \in 1, \ldots, k$, we have

$$\begin{aligned} 0 \geq m_{x,z_j}(T_a) - m_{x,z_j}(T_b) &= \left(\frac{\lambda}{1-\lambda}\right)(M_{x,z_j}(T_b) - M_{x,z_j}(T_a)) \\ &\geq \left(\frac{\lambda}{1-\lambda}\right)(M_{x,y}(T_b) - M_{x,z_j}(T_a)) \\ &= \left(\frac{\lambda}{1-\lambda}\right)(M_{x,y}(T_b) - M_{x,y}(T_a) + M_{x,y}(T_a) - M_{x,z_j}(T_a)) \end{aligned}$$

But $M_{x,y}(T_b) - M_{x,y}(T_a) = \left(\frac{1-\lambda}{\lambda}\right) n > 0$ and $M_{x,y}(T_a) - M_{x,z_j}(T_a) > 0$ so we have a contradiction. Thus Equation 1 cannot hold for all $i, j \in \{1, \ldots, k\}$, so $d_\lambda(T_a, T_b) = 0 \Rightarrow T_a = T_b$. □

1.2 The role of λ

The parameter λ allows the user to choose to what extent the branch lengths of a tree, versus its topology alone, contribute to the tree distance. This is useful in applications in which the topology of the tree is relatively well-defined by data but where the root height is difficult to infer, as can be the case in coalescent analyses. Here, trees may appear close topologically ($\lambda = 0$) but more distant when lengths

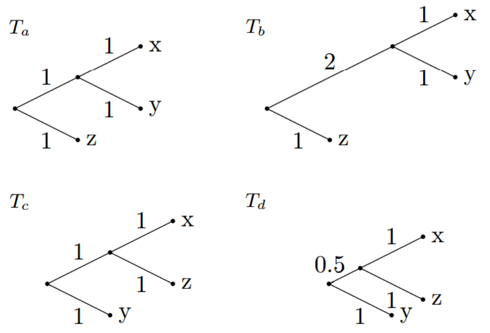

$$
\begin{aligned}
v_\lambda(T_a) &= (1-\lambda)(1,0,0,1,1,1) + \lambda(1,0,0,1,1,1)\\
v_\lambda(T_b) &= (1-\lambda)(1,0,0,1,1,1) + \lambda(2,0,0,1,1,1)\\
v_\lambda(T_c) &= (1-\lambda)(0,1,0,1,1,1) + \lambda(0,1,0,1,1,1)\\
v_\lambda(T_d) &= (1-\lambda)(0,1,0,1,1,1) + \lambda(0,0.5,0,1,1,1)
\end{aligned}
$$

$d_\lambda =$

	T_a	T_b	T_c
T_b	λ		
T_c	$\sqrt{2}$	$\sqrt{\lambda^2+2\lambda+2}$	
T_d	$\sqrt{\frac{1}{4}\lambda^2-\lambda+2}$	$\sqrt{\frac{5}{4}\lambda^2+\lambda+2}$	$\frac{1}{2}\lambda$

Figure 2: Example trees from $\mathcal{T}_3$ to illustrate the effect of changing λ. The distance between T_a and T_c ($d_\lambda(T_a, T_c)$) is fixed for $\lambda \in [0,1]$ because their unmatched edges have the same length. $d_\lambda(T_b, T_d) < d_\lambda(T_b, T_c)$ for $\lambda \in (0,1]$ because the edge which T_c and T_d share and which is not found in T_b is shorter in T_d than in T_c. Most entries increase with λ. The only distance to decrease as $\lambda \to 1$ is $d_\lambda(T_a, T_d)$, because the difference between the lengths of their unmatched branches is less than one.

are considered, and the length-based analysis will identify genes with different root heights whereas the topological analysis will compare tree structures. In general, the distance between two trees may increase or decrease as λ increases from 0 to 1. Since the topology-based vector, m, contains the number of edges along paths in the tree, and M contains the path lengths, the branch lengths are implicitly compared to 1 in the convex combination v_λ. In other words, if the branch lengths are much larger than 1, then the entries of M will be much larger than the corresponding entries of m, and M will dominate in the expression for v_λ even when λ is relatively small. Conversely, if the branch lengths are much less than 1, the entries of M will be much less than those of m, and a value of λ near 1 will be required in order for lengths to substantially change v_λ. In the case when all branch lengths are equal to 1, $m = M$ and the distance is independent of λ. The example in Figure 2 may provide some intuition.

Appropriate choice(s) of λ will depend on the focus of the analysis and the interpretation of the branch lengths, which can correspond to times or rates and will of course vary in magnitude depending on the units (e.g. substitutions per site per year versus substitutions per site per day.) In order to capture length-sensitive distances between trees, we may wish to use a value of λ such that neither $(1-\lambda)m$ nor λM dominate excessively, but naturally this will depend on the analysis. For a more gradual change in d_λ as λ tends to 1, and for comparison of this change across different data sets, it is possible to rescale the branch lengths, for example by dividing all branch lengths by the median, or by changing the units. However, this should be done with caution because information is inevitably lost through rescaling. For example, if a phylogenetic analysis of multiple genes from the same organism had produced trees with similar topologies but different clock rates (e.g. branches in trees from gene 1 were typically twice as long as branches in trees from gene 2), this information would be obscured by rescaling.

1.3 Further properties and extensions of our metric

Our metric is fundamentally for *rooted* trees. A single unrooted tree, when rooted in two different places, produces two distinct rooted trees, and our distance between these will be greater than zero. It will be large if the two distinct places chosen for the roots are separated by a long path in the original unrooted tree. However, it would be straightforward to check if two trees have the same (unrooted) topology in our metric: root both trees on the pendant edge to the same tip and find the distance. Re-rooting a tree will induce systematic changes in $v_\lambda(T)$, with some entries increasing and others decreasing by the same amount.

The metric d_λ is invariant under permutation of labels. That is, for trees T_a and T_b and a label permutation σ, $d_\lambda(T_a, T_b) = d_\lambda(T_a^\sigma, T_b^\sigma)$, since this operation corresponds to permuting the entries of each vector in the same way.

We note that alternative, similar definitions for a metric on $\mathcal{T}_k$ are possible. In particular, the metric defined by

$$D_\lambda(T_a, T_b) = (1-\lambda)\|m(T_a) - m(T_b)\| + \lambda\|M(T_a) - M(T_b)\|$$

gives similar behavior to the metric we have used. The difference between the two is that in D, the Euclidean distances are taken between the m and M vectors *before* they are weighted by λ rather than

after. Rather than a Euclidean distance between two vectors (v for each tree) as in the main text, D is a weighted sum of two different metrics: the distance between $m(T_a)$ and $m(T_b)$ (first term in the above), and between $M(T_a)$ and $M(T_b)$ (second term). A benefit of D_λ is that it is linear in λ, so that the changes as λ moves from 0 to 1 are more intuitive. A disadvantage is that D_λ itself is not Euclidean, leading to (typically only slightly) poorer-quality MDS plots.

As defined here, our metric compares trees with the same set of taxa (i.e. the same tips). As a consequence, it is suited to the kinds of questions we have described, in which there is one set of taxa and a collection of trees are to be compared from different genes, inference methods, and/or sources of data. However, in some cases, some data sources may not have data for all taxa (for example, not all genes may be present in all taxa). There are several natural extensions to our approach which allow comparisons of trees with not-quite-matching tip sets. For example, one can prune the trees until their tip sets match, optionally adding a 'penalty term' for each tip which has to be removed. Alternatively, placement of missing tips could be imputed using the quartet method of Holland et al. [Holland et al., 2007], nearest-neighbour methods or other tools from statistical treatments of missing data.

Similarly, there are natural extensions to comparing trees with (some) internal node labels, such as trees containing fossils as generated by the fossilised birth-death process [Heath et al., 2014]. Where there is no natural link between the labels of trees, comparisons between *unlabelled* trees (e.g. kernel methods [Poon et al., 2013] and spectral methods [Lewitus and Morlon, 2015]) are suitable.

The fact that our metric is a Euclidean distance between two vectors whose components have an intuitive description means that simple extensions are straightforward to imagine and to compute. For example, it may be the case that the placement of a particular tip or clade is a key question. This could occur, for example, in a real-time analysis of an outbreak, where new cases need to be placed on an existing phylogeny to determine the likely source of infection. We can form a metric that emphasises differences in the placement of a particular tip or set of tips (say, $t_1, \ldots, t_i$), by weighting $t_1, \ldots, t_i$'s entries of m and M highly compared to all other entries. In this new comparison, trees would appear similar if their placement of $t_1, \ldots, t_i$ was similar; patterns of ancestry among the other tips would contribute less to the distance. This functionality is implemented within *treescape* [Jombart et al., 2015].

1.4 Visualisation with MDS

Visualisation techniques like MDS have been used to explore tree space using other metrics [Holmes, 2006, Chakerian and Holmes, 2012, Amenta and Klingner, 2002, Hillis et al., 2005, Berglund, 2011] but have been challenged by poor-quality projections. When a multidimensional set of distances is projected into a low-dimensional picture there is typically some loss of information which may result in a poor-quality visualisation. For example, if 10 points are all 4 units away from each other this will not project well into two dimensions; some will appear more closely grouped than others. However, if there are only 3 such points they can be arranged on an equilateral triangle, capturing the distances in two dimensions. One approach to checking the quality of a visualisation is a Shepard plot [Shepard et al., 1972], which is a scatter plot of the 2- or 3-dimensional MDS distance versus the true distance from the metric. We include Shepard plots as insets in our supplementary figures to demonstrate their quality.

1.5 Navigating islands and selecting summary trees

Tree inference methods use data to constrain the set of possible trees to a relatively small region of tree space. The fact that data may support trees in separated regions or 'islands' of tree space has deep implications for tree inference and analysis [Maddison, 1991, Salter and Pearl, 2001]. A further complicating factor is that when taxa have incomplete data at some loci there can be 'terraces' of many equally likely trees, with trees in a terrace all supporting the same subtrees for the taxa with data at a given locus [Sanderson et al., 2011]. However, the difficulty of detecting and interpreting tree islands has meant that the majority of analyses, particularly on large datasets, remain based on a single summary tree. This may be a maximum clade credibility (MCC) tree with posterior support values illustrating uncertainty, or a maximum likelihood or parsimony tree with bootstrap supports [Heled and Bouckaert, 2013].

Our approach includes a natural way to group trees into clusters. Since distance is defined by the metric that is used, these are different from previously described tree islands [Maddison, 1991, Salter and Pearl, 2001]. We note that islands are of particular concern for tree inference and for outcomes that require the topology of tree such as ancestral character reconstruction; consequently they affect the interpretation of many phylogenetic datasets [Sullivan et al., 1996]. However, other analyses, and tree estimation methods themselves, take trees' branch lengths as well as topology into account. We find that

the clusters typically merge together in the metric as λ approaches 1; the posterior becomes unimodal (Figure S4).

There are many challenges to summarising complex tree spaces [Heled and Bouckaert, 2013]. Maximum clade credibility (MCC) trees are used to summarise posterior distributions by collecting the clades with the strongest posterior support. However, where these are not concordant the MCC tree can have negative branch lengths. Furthermore, the MCC tree itself may never have been sampled by the MCMC chain, casting doubt on its ability to reflect the relationships in the data.

In the main text we used the familiar method of MCC trees with posterior support values to demonstrate the way that each cluster corresponds to a possible, likely resolution of uncertain clades. In fact, we can also use the metric directly to find 'central' trees within any collection of trees using barycentric methods such as the geometric median [Haldane, 1948]. That is, we can exploit the fact that our metric is simply the Euclidean distance between the two vectors $v_\lambda(T_a)$ and $v_\lambda(T_b)$. Among N trees $T_1, \ldots, T_N$ in a posterior sample, we can find the tree closest to the average vector $\bar{v}_\lambda = \frac{1}{N}\sum_{i=1}^{N} v_\lambda(T_i)$. The average vector $\bar{v}_\lambda$ may not in itself represent a tree, but we can then find the tree vector(s) from our sample closest to this average - those which achieve the minimum distance between $\bar{v}_\lambda$ and each vector $v_\lambda(T_i)$. Each of these corresponds to an actual tree T_c from the original sample, with non-negative branch lengths. The minimal distance between the central vector and closest tree vectors is a measure of the quality of the summary: if it is small, each T_c is close to 'average' in the posterior. Such a tree T_c is known as the geometric median tree. Geometric median trees will always have been sampled by the MCMC, and will not have negative branch lengths. It is also straightforward to weight trees by likelihood or other characteristics when finding the geometric median. We found that within clusters, geometric median trees are very close (typically identical) in topology to the MCC tree for the cluster, but with differing, credible branch lengths.

There are several tests for comparing the support for different tree topologies in a maximum likelihood framework, including the KH [Kishino and Hasegawa, 1989], SH [Shimodaira and Hasegawa, 1999] and AU tests [Shimodaira, 2002]. However, these likelihood ratio tests are not applicable to our Bayesian framework. Figure 3B (main text) demonstrates that the log-likelihoods of the different clusters are comparable, with no single cluster dominating the others in likelihood. (Note that the actual likelihoods, rather than the log-likelihoods, are so similar as to be indistinguishable on such a boxplot.) It is therefore advisable to retain the full Bayesian posterior set of trees wherever practical. Where a small number of trees is required for further analysis, it is important to retain at least one summary tree for each cluster until alternative topologies can be ruled out by further data and analysis.

2 Relationship to other tree comparisons

Tree comparisons have been proposed for a variety of purposes. We provide a brief review of some of the existing tree comparison approaches in the literature and explain their relationships to our method.

2.1 Other metrics on labelled trees

Various metrics have been defined on phylogenetic trees [Kuhner and Yamato, 2014]. Table 1 provides a brief comparison of the characteristics of some existing metrics.

The vector $M(T)$ is similar to the cophenetic vector of Cardona et al. [Cardona et al., 2013], following Sokal and Rohlf [Sokal and Rohlf, 1962], where $M_{i,j}$ is called the *cophenetic value* of tips i and j. Parts (1) and (2) of our proof (Section 1.1) follow directly from results in Cardona et al. [Cardona et al., 2013]. Instead of the pendant branch lengths p_i, Cardona et al. use the depth of each taxon, which can be considered as $M_{i,i}$. This involves a repetition of information between $M_{i,i}$, $M_{j,j}$ and $M_{i,j}$ whenever $M_{i,j} > 0$. In fact, the final k entries of M are only required to distinguish between trees in the rare event that two trees have the same topology, identical internal branch lengths, but differences in their pendant branch lengths. Our rationale for using pendant branch lengths rather than tip depths was therefore to test for this variation without inflating tree distances by re-counting internal branch lengths. Indeed, since the final k entries of m are not required for our distance to be a metric when $\lambda = 0$, it is natural to choose them so that they have no effect on the distance (all equal to 1) rather than having them vary by tip depth. The two definitions clearly measure very similar properties of the trees, and for large k the first $\binom{k}{2}$ vector entries will dominate the tree distance. Experiments on a variety of random trees and trees inferred from data showed that the two definitions are highly correlated for each value of $\lambda \in [0, 1]$: the lowest Spearman correlation found was 0.89 for $k = 4, \lambda = 1$, with correlations of ≈ 0.99 for $k > 20, \lambda \in [0, 1]$. However, the symmetries of the space are slightly distorted when tip depths are

Metric	Root	Topology	Lengths	Euclidean	Convex	Computation
RF [1]	n	y	y	n	n	0.04
Branch score [2]	n	n	y	y	n	0.04
BHV [3]	y	n	y	n	y	30.78
Path (l^1-norm) [4]	n	y	y	n	n	94.97*
Path (l^2-norm) [5]	n	y	y	y	n	0.04
Quartet [6]	n	y	n	n	n	$\mathcal{O}(n \log n)$ [7]
Triplet [8]	y	y	n	n	n	$\mathcal{O}(n \log n)$ [9]
rooted SPR [10]	y	y	n	n	n	NP-hard [11]
Ours	y	y	y, flexibly	y	n	0.2

Table 1: A table to compare the applicability of various metrics. The metrics chosen here are applicable to pairs of trees with the same tip labels. We note that variations of these metrics have been proposed in the literature and so it may be possible that variants exist with slightly different properties. Here we compare to the best of our knowledge the metrics as originally proposed in the papers cited. We compare: *Root:* does the metric use the position of the root? *Topology:* does the metric (or a version of it) use topology only? *Lengths:* does the metric (or a version of it) use the branch lengths? *Euclidean:* are the distances Euclidean? *Convex:* is the metric convex? *Computation:* an indication of the computational complexity. Where an R implementation is available we present the system time in seconds taken to compare a pair of trees each with 1000 tips, on the same computer (Intel(R) Core, i3-2370M CPU, 2.40 GHz, 4GB RAM). The functions used were *RF.dist* from *phangorn* [Schliep, 2011] for RF, *KF.dist* from *phangorn* [Schliep, 2011] for branch score distance, *dist.multiPhylo* from *distory* [Chakerian and Holmes, 2013] for BHV, *distTips* from *adephylo* [Jombart and Dray, 2010] for the l^1-norm path difference, *path.dist* from *phangorn* [Schliep, 2011] for the l^2-norm path difference, and *treeDist* from *treescape* [Jombart et al., 2015] for our metric. *The time for the l^1-norm path difference could be substantially improved to match that of the l^2-norm but we did not find such an implementation.

used: Figure 3 revisits our example from the main text on the trees with six tips (Figure 2A, $\lambda = 0$) with the alternative definition of the metric, using tip depths rather than pendant lengths for the final k entries of each tree vector. The 3-fold symmetry is less apparent, particularly in the 'pink' trees, and the more balanced trees are less central. Nevertheless, much of the intuitive structure remains, and a key advantage of the tip depth approach of Cardona et al. is that it allows for the presence of nested taxa (taxa which are internal nodes of the tree).

We have compared our metric to that of Robinson and Foulds (RF) [Robinson and Foulds, 1981] in the main text because it is the most widely used metric. However, RF and its branch-length weighted version [Robinson and Foulds, 1979] are fundamentally different from our metric because they are defined on unrooted trees, whereas our metric emphasises the placement of the root and all the descendant MRCAs. Similarly, many other metrics are designed for unrooted trees, including the branch score distance [Kuhner and Felsenstein, 1994] and the tip-to-tip path length metrics of Williams and Clifford [Williams and Clifford, 1971] (using the l^1-norm) and Steel and Penny [Steel and Penny, 1993] (using the l^2-norm). These tip-to-tip path metrics can either be weighted by branch lengths or not. In common with much of the literature we will refer to the Steel and Penny metric as the 'path difference metric' but note that it also goes by many other names including the patristic distance, nodal distance, tip distance and dissimilarity measure. The path difference metrics compare the distance between each pair of tips in a tree; in essence, they consider the distance between *tips* and their MRCA, whereas our metric considers the distance between the *root* and the MRCA. The branch score distance is closely related to the RF metrics [Kuhner and Felsenstein, 1994]: unweighted RF counts the unmatched edges between trees, weighted RF sums the branch lengths of unmatched edges, whereas the branch score dis-

[1] [Robinson and Foulds, 1979, Robinson and Foulds, 1981]
[2] [Kuhner and Felsenstein, 1994]
[3] [Billera et al., 2001]
[4] [Williams and Clifford, 1971]
[5] [Steel and Penny, 1993]
[6] [Estabrook et al., 1985]
[7] [Brodal et al., 2013]
[8] [Critchlow et al., 1996]
[9] [Brodal et al., 2013]
[10] [Hein et al., 1996]
[11] [Bordewich and Semple, 2005]

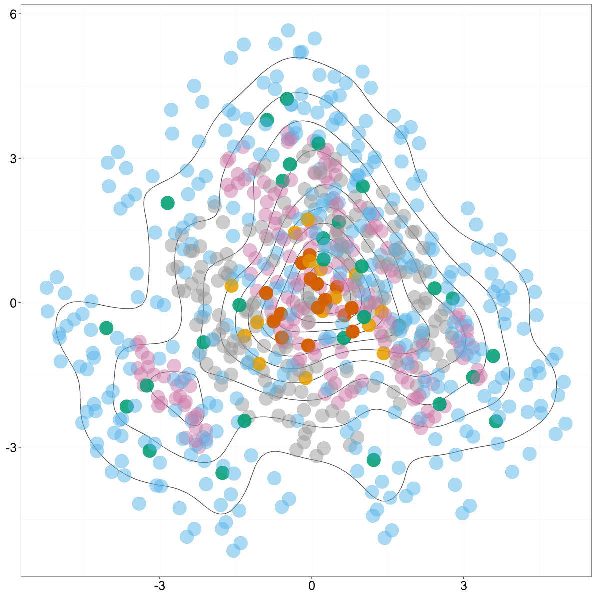

Figure 3: The space of trees with six tips according to an alternative definition of the metric where the final k entries are the tip depths rather than pendant lengths ($\lambda = 0$). In parallel to Figure 2A, the colours correspond to tree shapes.

tance takes the sum of squared differences between matched internal branch lengths, plus the squared lengths of unmatched branches. For a pair of trees where all branches have length 1, these three measures give the same distance [Kuhner and Felsenstein, 1994]. The version of the branch score distance which we use, from the R implementation in *phangorn* [Schliep, 2011], calculates the square root of the measure described above (as defined later in [Kuhner and Felsenstein, 1994]), which is a metric.

The metric introduced by Billera, Holmes and Vogtmann (BHV) captures branch lengths as well as tree structure [Billera et al., 2001] on rooted trees. The BHV tree space is formed by mathematically 'glueing' together orthants. Each orthant corresponds to a tree topology and moving within an orthant corresponds to changing the tree's branch lengths. Moving from one orthant to an adjacent one corresponds to a nearest-neighbour interchange move. The metric is convex: for any two distinct trees T_1 and T_2, there is a tree T_3 'in between' them, i.e. such that $d_{BHV}(T_1, T_3) + d_{BHV}(T_3, T_2) = d_{BHV}(T_1, T_2)$. This is a mathematically appealing and useful property, in part because it allows averaging of trees [Bacak, 2014]. The metric does not allow the user to choose a balance between the topology of the tree and the branch lengths.

We have included the rooted SPR distance [Hein et al., 1996] in Table 1 since it is most natural to compare our metric to other rooted metrics. We note that there is also a definition for SPR on unrooted trees, but this is extremely difficult to calculate. Recent work [Whidden and Matsen IV, 2015] has enabled the computation of unrooted SPR distances as large as 14 on trees with 50 tips.

As noted in the main text, any positive linear combination of metrics is a metric, so tree metrics can be combined as desired to detect a variety of features within a tree comparison [Liebscher, 2015]. For example,

$$d^*(T_a, T_b) := w_1 d_0(T_a, T_b) + w_2 d_{RF}(T_a, T_b) + w_3 d_{BHV}(T_a, T_b)$$

where $w_1, w_2, w_3 > 0$ are weight coefficients, would be a metric which compares trees from both rooted and unrooted perspectives, with a contribution from the branch lengths in the d_{BHV} term. Cardona et al. also note that vectors which characterise trees can be compared by any norm l^p, but that the Euclidean norm l^2, which we also use, has the benefits of being more discriminating than larger values of p, and enabling many geometrical and clustering methods.

2.2 The set of trees with six tips

Figure 4 shows the MDS plot of the space of trees on six tips (with unit branch lengths) under our metric, as in the main text. For comparison, we also provide the analogous plots according to other metrics, namely RF [Robinson and Foulds, 1981], BHV [Billera et al., 2001] and the path difference metric [Steel and Penny, 1993]. Note that because we have used unit branch lengths, $m = M$ and so our

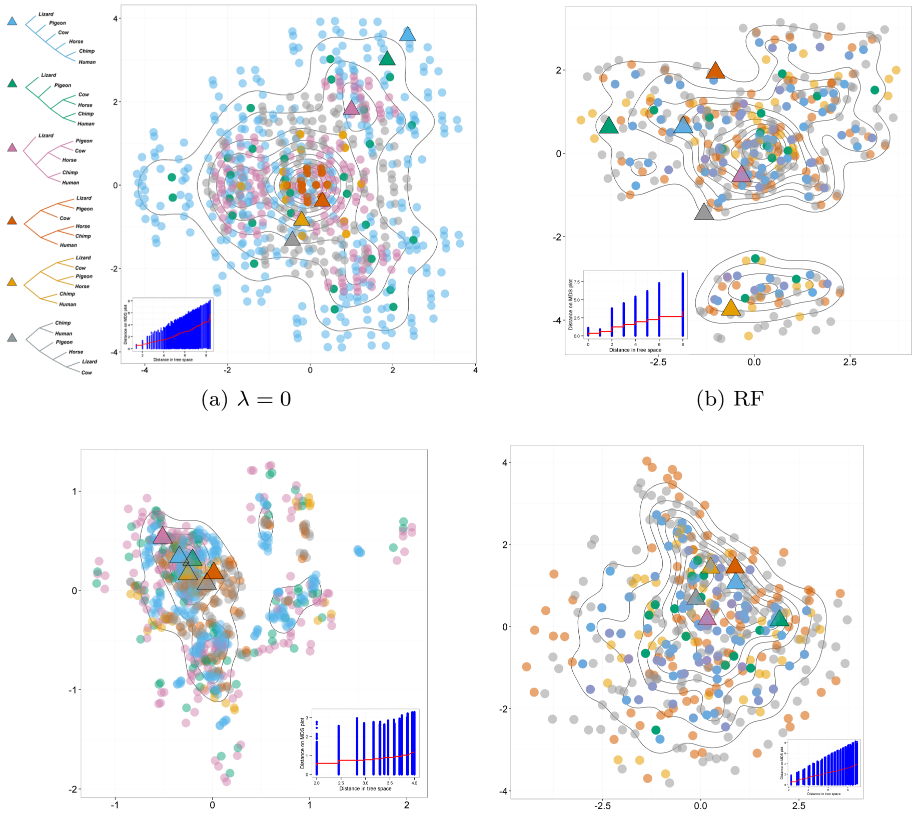

(a) $\lambda = 0$ (b) RF

(c) BHV (d) Path difference

Figure 4: MDS projections of the shape of $\mathcal{T}_6$ according to various 'topological' metrics, with corresponding Shepard plots. In order to include the BHV metric in this comparison we assigned all branch lengths to be 1, with the result that on these trees, our metric is invariant to $\lambda \in [0, 1]$ and the unweighted and weighted RF metrics are the same.

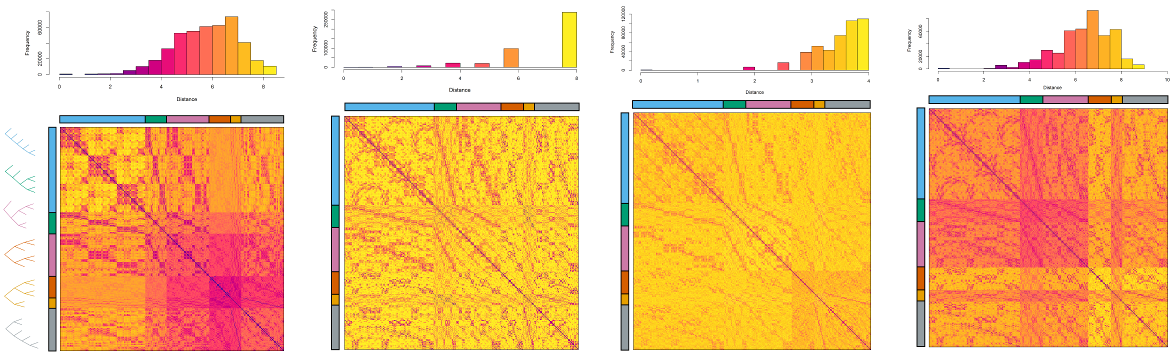

(a) Our metric; $\lambda = 0$ (b) RF (c) BHV (d) Path difference

Figure 5: Histograms and matrices to show the distances between all 945 trees on six tips, according to different tree metrics. Colours along the vertical and horizontal axes correspond to the shape of tree, as in Figure 4a.

metric is invariant under $\lambda \in [0, 1]$ in this example. Similarly, the weighted and unweighted RF metrics will give the same distances for these trees, and the branch score metric would simply give the square root of those distances. There is no 'topology only' version of the BHV metric, but applying it to trees with unit branch lengths provides a natural comparison to the 'topological' metrics (metrics which disregard branch lengths).

All 945 possible tree shapes and permutations of their labels are present in the input set of trees, and consequently there is no asymmetry that should lead to one group being separated from the rest. Our

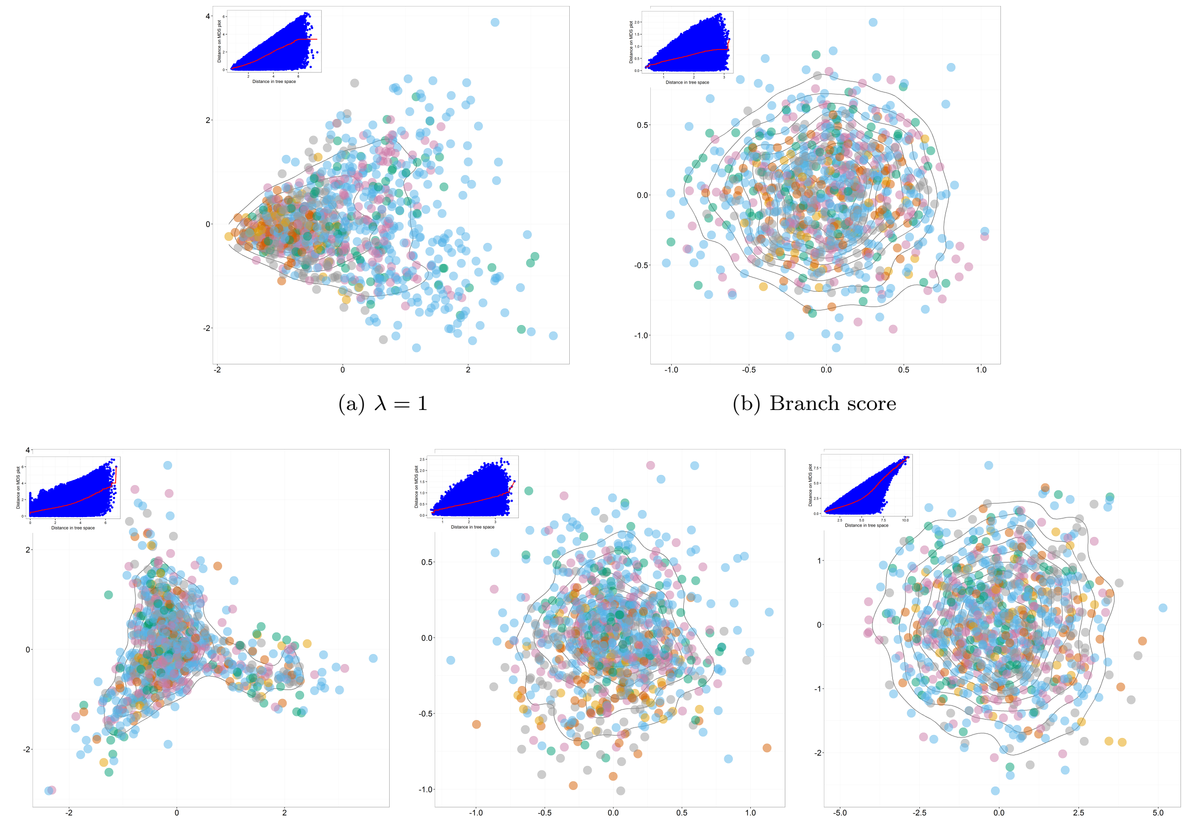

(a) $\lambda = 1$ (b) Branch score

(c) Weighted RF (d) BHV (e) Weighted path difference

Figure 6: MDS projections of the trees in $\mathcal{T}_6$ with random branch lengths according to various branch-length metrics, with corresponding Shepard plots.

metric captures the symmetry in the space and illustrates this in the MDS projection (Figure 4a). The Euclidean nature of our metric means that it is well-suited to visualisations that project distances into two- or three-dimensional Euclidean space. In contrast, in the RF and BHV metrics (Figures 4b and 4c), poor-quality projections lead to apparent distinct tree islands where none exist. This makes detecting genuine islands in posterior sets of trees difficult using RF or BHV.

The inset Shepard plots (see Section 1.4) are a way to assess the quality of an MDS projection. They illustrate that the correspondence between the projected distances and true distances is better in our metric than RF and BHV, and approximately as good as the path difference metric, though the projection distance can be much smaller than the true distance (but not the converse). MDS projections are of higher quality for trees from data than in the space of all trees on six tips (e.g. Figure 9).

The path difference metric (Figure 4d) shows some symmetry and structure. The more balanced tree shapes naturally occur towards the extremes of the space, as they achieve the largest tip-tip distances. In contrast, in our metric highly unbalanced trees appear near the edges of the space because the MRCAs can achieve greater distances from the root, and because permutations of the tips on unbalanced trees lead to greater differences in the root to MRCA distances than permutations of tips in more symmetric trees. This illustrates that the path difference metric measures and prioritises different tree characteristics from ours.

To supplement Figure 2B from the main text, we repeat the histograms and distance matrices on the space of all trees with six tips for our metric and RF, and additionally provide the histograms and distance matrices for BHV [Billera et al., 2001] and the path difference metric [Steel and Penny, 1993] in Figure 5. The spreads of the histograms show that BHV and the path difference detect more subtle differences than RF. Distinctions between tree shapes in the distance matrix are more noticeable in the path difference than in RF or BHV, and are most clearly distinguishable in ours.

We provide in Figure 6 an analogue to Figure 4 using the branch-length sensitive metrics on the six-tip trees, where each tree was assigned random branch lengths sampled uniformly at random in the range $(0, 1)$. This toy example serves to show that, typically, projections are of better quality when branch lengths are included (inset Shepard plots) than when comparing trees by topological metrics.

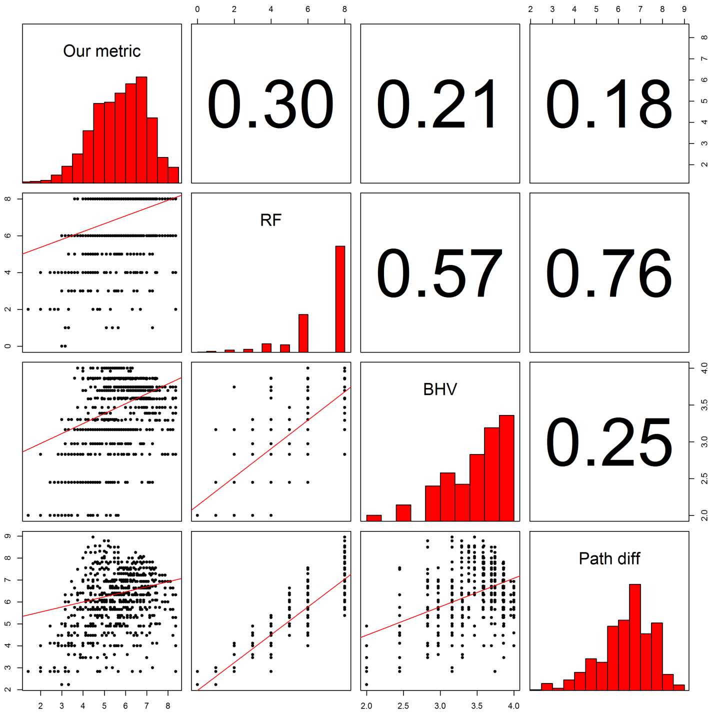

Figure 7: Panel of scatterplots and Spearman correlation coefficients to show the relationships between the topological tree metrics implemented in R: our metric (top left, $\lambda = 0$), unweighted RF, BHV (trees with unit branch lengths) and unweighted path difference (Steel & Penny).

This is largely explained by the fact that the branch-length metrics each produced a range of values (see histograms in Figure 8), resulting in distances which are easier to project than collections of nearly-equidistant points. The Shepard plot for the weighted path difference shows that it lends itself particularly well to 2D-projection in this example. We have found that points (trees) tend to be much closer together according to branch-length metrics than with topological metrics, and that large 'gaps' between tree topologies are usually masked when branch lengths dominate. The weighted RF metric (Figure 6c) is something of an exception in this respect, presenting more of a '3-arm' structure than a single cluster. The colours in these plots correspond to the tree shapes as before. We see that tree shape information is largely masked by branch lengths, although the property of pectinate trees achieving the largest distances from more balanced trees is preserved to a small extent in some of the plots.

Finally, in Figures 7 and 8 we present a comparison of the distances given by each of the tree metrics implemented in R. For Figure 7 we sampled 5000 of the 446040 possible pairwise tree comparisons on all 945 trees with six tips, and present scatterplots of the distances according to each metric. The Spearman's rank correlation coefficient between our metric and others is weak; the strongest correlation we found (0.76) is between RF and the path difference metric, but it is clear that each of these metrics measures different properties of trees. For Figure 8 we used the six-tip trees with random branch lengths. We again sampled 5000 of the 446040 possible pairwise tree comparisons using five branch-length metrics: our metric ($\lambda = 1$), weighted RF (which we implemented based on 'RF.dist' and 'KF.dist' from *phangorn*), branch score distance, BHV and weighted path difference. Here, the most strongly correlated metrics are branch score distance with BHV (0.73) and weighted path difference (0.74), but again we see that each metric prioritises different tree characteristics.

2.3 Other tree comparison methods

Comparing phylogenetic trees, and comparing the quality with which they capture a set of data, are long-standing challenges for which there are several approaches. The KH [Kishino and Hasegawa, 1989], SH [Shimodaira and Hasegawa, 1999] and AU tests [Shimodaira, 2002] use classical hypothesis testing to compare the likelihoods of a given set of data across different phylogenetic trees. These tools are suited either to topologies chosen *a priori* and not derived from the same data set (in the case of the KH test), or

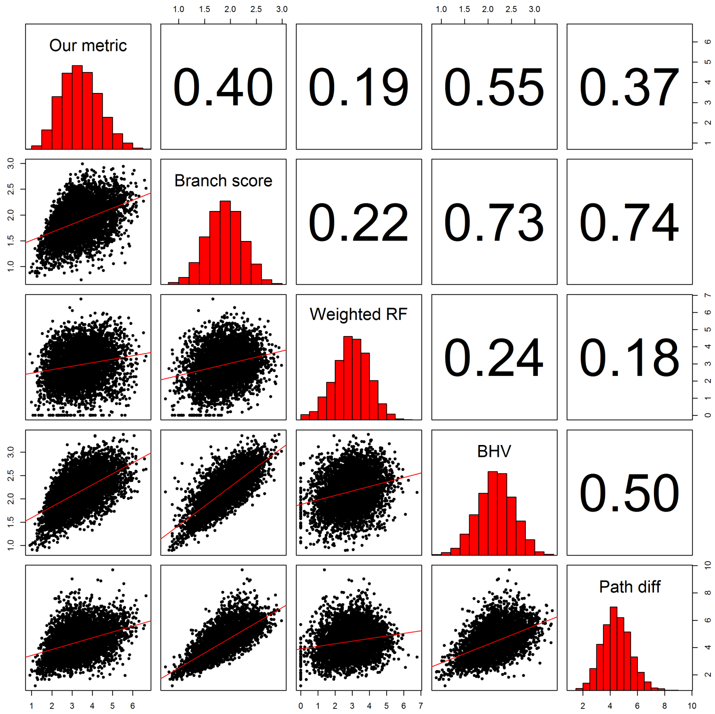

Figure 8: Panel of scatterplots and Spearman correlation coefficients to show the relationships between the branch-length tree metrics implemented in R: our metric (top left, $\lambda = 1$), branch score distance (strongly correlated with weighted RF), BHV and weighted path difference (Steel & Penny).

to tree quality comparison in a maximum likelihood setting. They do not capture the diversity, structure and distribution of a set of trees themselves, and do not naturally map to the Bayesian setting. In the Bayesian setting, the posterior collection of trees represents the collection of trees one should consider given the data and the prior; it is not appropriate to use likelihood-based tests to select from the posterior. If obtaining a maximum likelihood tree is the aim, then maximum likelihood tree inference tools should be used directly. In this case, visualising and comparing trees derived from bootstrapping is a natural application of our metric-based approach.

Many efforts to compare trees directly to each other have used the RF metric or closely-related partition/split comparisons and similarity measures. These tools include:

- MDS plots [Amenta and Klingner, 2002, Hillis et al., 2005, Berglund, 2011] of RF distances between trees
- kdetrees [Weyenberg et al., 2014] (which uses either RF, BHV geodesic distances or path difference; see below)
- Nye et al.'s alignment similarity measure [Gilks et al., 2006]
- Koonin et al.'s work on the forest of life [Koonin et al., 2011]
- CONCLUSTADOR [Leigh et al., 2008] and CONCATERPILLAR software [Leigh et al., 2011]
- Nye's use of Robinson-Foulds distances to create trees of trees [Nye, 2008]
- Chaudhary's extension of RF to multi-labelled trees [Chaudhary et al., 2013].

Accordingly, these comparisons may suffer in performance because of the limitations of the RF metric. For example, Koonin et al. [Koonin et al., 2011] compared the fractions of splits in common between trees to chart the 'forest of life', and commented that 'nearly universal trees' were grouped within a cluster (of trees) and nearly equidistant from other clusters. We have found that the RF metric may frequently produce equidistance, and that RF clustering does not necessarily reflect intuitive tree relationships. Leigh et al. [Leigh et al., 2011] noted that the Conclustador software may have erratic behaviour due to

large RF distances resulting from small rearrangements, and Hillis et al. [Hillis et al., 2005] noted the poor quality of MDS projections of RF tree-tree distances, which arises in part because the RF metric takes on relatively few different values.

Other tree comparison approaches which do not rely on the RF metric or the fraction of split differences include:

- MDS plots of BHV distances [Holmes, 2006, Chakerian and Holmes, 2012]
- kdetrees [Weyenberg et al., 2014] using the BHV or path difference metrics
- Phylo-MCOA [de Vienne et al., 2012]
- finding clusters of similar phylogenies amongst gene trees [Gori et al., 2016]
- the Maximum Agreement SubTree (MAST) method [Finden and Gordon, 1985]
- methods relying on MDS, PCA or MCOA projections of tree distances [de Vienne et al., 2012, Choi and Gomez, 2009]
- tests for incongruence amongst trees [Haws et al., 2012, Salichos et al., 2014]

The R package kdetrees [Weyenberg et al., 2014] aims to find 'outlier' trees using tree comparisons, and is best for datasets with a large number of genes, or sources of trees, and a relatively small number of taxa. In infectious disease applications, particularly for viruses, the situation is typically the opposite: a large number of taxa (thousands) and small number of genes (perhaps fewer than a dozen). Nevertheless, we compared kdetrees to our method using the kdetrees published example: a set of 268 trees, each with 8 tips. Our method identifies the same set of outliers, more quickly than kdetrees (0.33 secs versus 2.11 secs on a desktop computer). More fundamentally, though, trees identified as outliers are outliers because of their unusual branch lengths. A simple check of the mean branch lengths recovers the same outliers in just 0.03 seconds. At its heart, kdetrees suffers from the limitations of its underlying metrics: RF is not sufficiently sensitive and too many trees appear the same distance apart, and approaches like the BHV metric which cannot adjust for branch lengths are easily dominated by differences in lengths alone. In a larger test of 100 trees, each with 100 tips, kdetrees crashed whereas our function completed in 1.48 seconds.

Phylo-MCOA [de Vienne et al., 2012] also aims to find 'outlier' trees and relies heavily on PCO projections, which often do not capture distances well. Gori et al. use a variety of unrooted tree metrics followed by clustering methods to classify genes by common evolutionary history [Gori et al., 2016]. This technique is particularly appropriate for scenarios where there are many gene trees and a small number of phylogenetic clusters, but again carries the limitations of the underlying metrics. The MAST method [Finden and Gordon, 1985] is a similarity score based on the size of the maximum shared subtree. It is NP-hard to compute and has been outperformed by the RF metric in simulation experiments [Kuhner and Yamato, 2014].

Several methods have relied on MDS, PCA or MCOA projections of tree distances to compare trees [de Vienne et al., 2012, Choi and Gomez, 2009]. Directly comparing distance projections of trees allows trees of different sizes to be compared, but this is at the cost of comparing projections which are potentially very poor-quality reflections of the underlying structure in trees. In contrast, as described in Section 1.3, natural extensions of our metric and others can permit metric-based comparisons of trees with overlapping but non-identical tip sets, without the loss of information in the trees and without the need to align PCA-style projections to each other.

Haws et al. test for phylogenetic incongruence using a machine-learning approach which relies on *a priori* knowledge of dissimilarity between groups of trees [Haws et al., 2012]. Salichos et al. use information-theoretic ideas (Shannon entropy) together with partition frequencies to quantify tree incongruence [Salichos et al., 2014]. This approach is closely linked to maximum likelihood tree inference with bootstrap, and to the support values we have used in MCC trees. We have found that the more tightly an apparent cluster of trees is grouped in our metric, the higher the support values of the cluster's MCC tree (see Figure 11).

Although state of the art tools for recombination detection are based on direct sequence comparison and do not use trees [Croucher et al., 2015, Stenetorp et al., 2012] there are methods for detecting recombination which both construct trees and compare them. These are best suited to divergent data with relatively few taxa and genes. For example, the GARD software [Pond et al., 2006] is limited to at most 50 sequences on its web server, and the Boussau et al. models [Boussau et al., 2009] can be applied

to ‘dozens’ of sequences. They rely on neighbour-joining methods for their tree inference which means that they are unable to exploit the richer phylogenetic information obtained from more time-consuming inference methods such as BEAST. On our data, GARD produced a single neighbour-joining tree for the VP30 gene of Ebolavirus and found no evidence for recombination. The tree was different from any of those produced in the BEAST analysis from the same sequences. Neighbour-joining trees have the significant drawback that they often have negative branch lengths resulting from inter-taxa distance patterns that are not consistent with a tree.

Our approach detects phylogenetic incongruence, which can be a natural result of non-tree-like evolution such as recombination or hybridisation. Our tree metric could in principle be part of a pipeline to detect recombination, either by comparing trees from different genes, or by comparing trees derived from sequences with particular loci removed: if a locus, when removed, substantially alters an inferred tree it may be a sign that the locus contains different phylogenetic information than the rest of the data. We have not benchmarked methods based on the metric’s tree comparison against other tree-based approaches to detect recombination. Furthermore, detecting phylogenetic incongruence between gene trees using tree metrics has the limitation that genic data must contain sufficient variation to infer high-quality trees. Where this is the case, we suggest that constructing and comparing high-quality trees with the aid of informative tree metrics is a useful tool with which to explore data. Where genic data are not sufficient for high-quality tools or where recombinations are likely to occur within genes, other tools are likely most appropriate.

3 Supplementary results

3.1 Anole lizards

Figure 9 shows the MDS plots of posterior species trees for several values of λ, increasing from $\lambda = 0$ (as in the main text) to $\lambda = 0.1$ (where branch lengths are weighted quite highly because the lengths are often much larger than 1, with a mean of 13 and median of 3.7 in units of millions of years before the present). As λ increases, clusters spread and merge together, though at $\lambda = 0.1$ they remain visible as distinct ‘strips’, particularly in the 3D plot. The division between the left and right sides of the plot persists. Note that the tree colouring is the same on each plot to help see how the clusters ‘merge’; clustering algorithms typically do not continue to identify the same clusters as λ increases. Recall that the trees in Figure 3 of the main text are displayed as cladograms (branch lengths = 1) because we compared tree topologies ($\lambda = 0$) and this makes differences more clear. In Figure 10 we show the same trees with their original branch lengths. Many of the uncertain clade resolutions occur around short edges, which explains why the tree differences become less distinct as λ increases.

Individual gene trees in the anole data had large polytomies and did not mirror these alternative resolutions of the uncertainty in the posterior [Geneva et al., 2015], though it is possible that these alternatives would be mirrored by gene trees with a sufficiently high number of samples. It is also possible that there are clusters of likely tree topologies that the *BEAST algorithm did not reach.

The same approach (pairwise distances followed by k-means clustering) that we have used with our distances can, in principle, be used with any metric or quantitative tree comparison tool. We used k-means clustering and comparison of BIC values to cluster the anoles posterior species trees using RF distances. Figure 11 shows the results. k-means clustering will always allow some clusters to be obtained; we found that $k = 12$ groups minimised the BIC here.

Two of the RF clusters broadly correspond to the groups we identified (in the sense that the MCCs have the same topology), namely our orange cluster containing the posterior MCC, and our pale orange cluster. The RF clusters are not visibly tightly grouped or well-separated in either 2D or 3D MDS plots (Figures 11a and 11b). Shepard plots show that the correlation between the projected distances and the RF distances is not strong, so visible grouping is not a good test of how meaningful the clusters are. We compared the ‘tightness’ of clusters, measured by the mean tree-tree distances within clusters, to the level of certainty (posterior support values) in MCC trees for the clusters. Our metric has higher MCC support in tighter clusters. In other words, groups of trees a small distance from each other have more highly resolved clades than those far from each other (Figure 11d). The pink RF cluster is the only large, well-supported cluster that the RF metric detects and its topology is the same as the MCC. The other RF clusters have posterior support values that are more uncertain than the posterior itself. In other words, the RF metric does not resolve uncertainty into distinct, well-supported alternative trees (e.g. Figure 11c).
In contrast, our metric identifies large, tight clusters with high posterior supports.

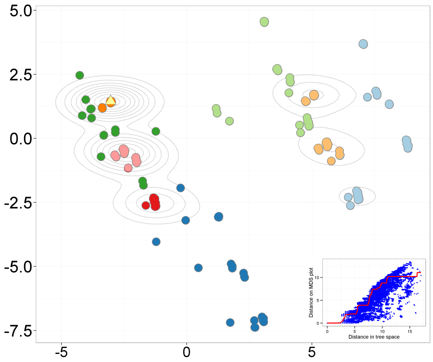

(a) $\lambda = 0$

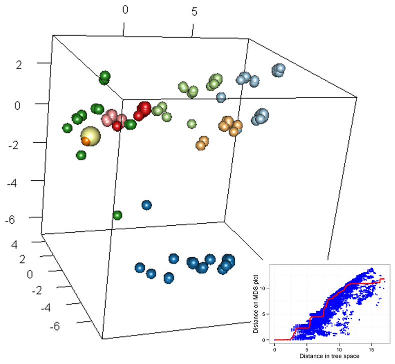

(b) $\lambda = 0$

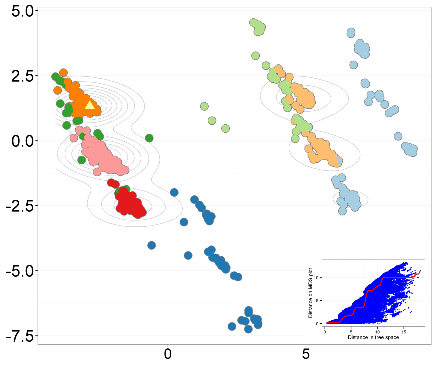

(c) $\lambda = 0.05$

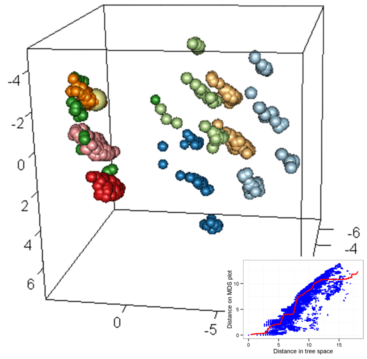

(d) $\lambda = 0.05$

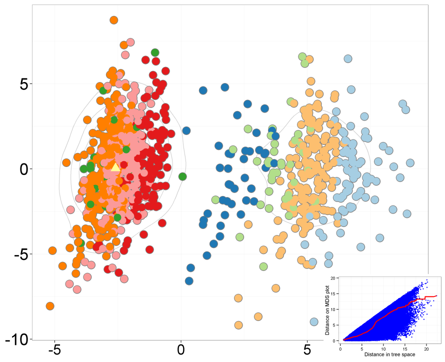

(e) $\lambda = 0.1$

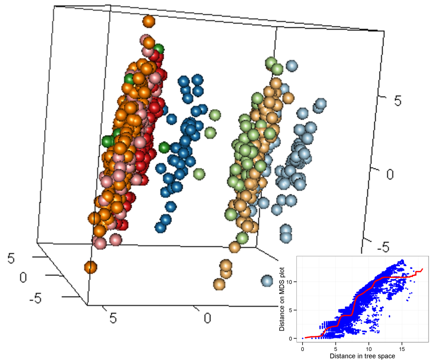

(f) $\lambda = 0.1$

Figure 9: MDS plots of the posterior anoles species trees for several choices of λ. (a) is the same as Figure 3C in the main text; (b) is a 3D MDS plot of the same clusters with the same colours, which better shows the separation, particularly between the dark blue cluster and the others. (c) and (d) are 2D and 3D MDS plots of the tree distances when $\lambda = 0.05$. The inclusion of lengths spreads the trees out; whereas in (a), all trees with the same topology are plotted on top of each other, here, variation in the branch lengths contributes to the distances, spreading the clusters out. (e) and (f) have $\lambda = 0.1$. Clusters are merging together somewhat, but are still distinctive, which the 3D visualisation illustrates. 3D plots have an additional degree of freedom, allowing MDS projected distances to be more closely correlated with the input distances than in 2D (inset Shepard plots).

When there are distinct, likely alternative topologies, we suggest that it is often not sufficient to retain only one tree for further analysis. In the case of the anole lizards, trees from different clusters support different conclusions about their biogeographic origins and dewlap colouration. Using the locations given in [Geneva et al., 2015] we plotted the geographical information given by our alternative trees using `phylo.to.map` from the R package `phytools` [Revell, 2012]. Two examples are provided in Figure 12. In parallel with Geneva et al., we have discarded the outgroup for these trees for ease of plotting. Results indicate that *ocior* and *distichus* (the anoles from the satellite islands and Bahamas) are more

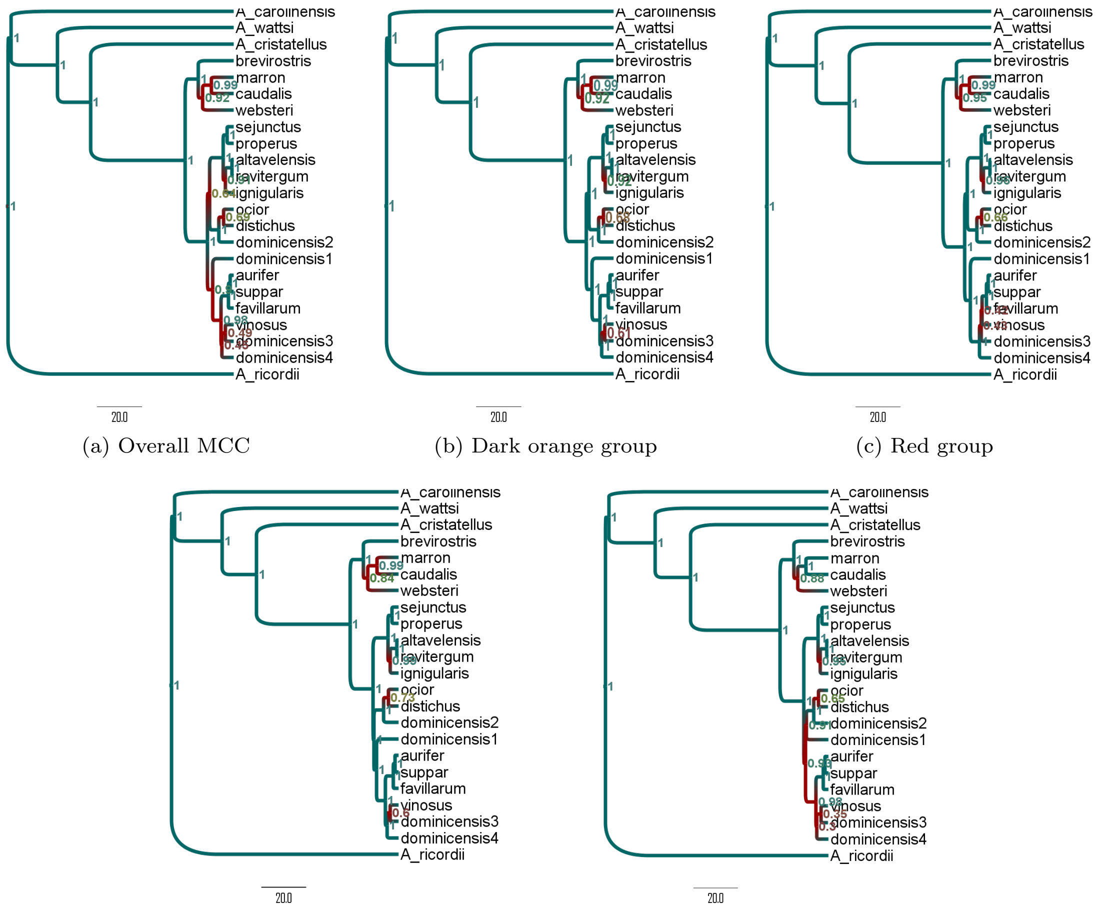

(a) Overall MCC (b) Dark orange group (c) Red group

(d) Light orange group (e) Light green group

Figure 10: Anoles consensus trees from our $\lambda = 0$ analysis, showing lengths in millions of years.

closely related to anoles from the East of Hispaniola (the North paleo-island) according to the MCC tree (Figure 12a) but to anoles from the South-West (the South paleo-island) according to other likely trees (Figure 12b).

We also performed stochastic character mapping to explore the evolution of dewlap colouration. Following Geneva et al. [Geneva et al., 2015], we discarded the outgroup and added duplicate tips for *caudalis* and *favillarum* which are found with both pale yellow and dark orange/red dewlap colours. Using the dewlap colours from [Geneva et al., 2015] we performed an MCMC exploration of possible transition matrices using `make.simmap` from `phytools`, sampling 1000 times. Whilst all eight tree topologies supported the conclusion that evolution between the colours has occurred repeatedly across the species group, the inferred transition rates differed. The posterior stochastic character maps for each tree topology varied dramatically (with the root node prior probabilities being 0.5 each for yellow and red), showing for example different colourings for ancestors of the major clades. However, given high transition rates the internal colouring is difficult to infer with confidence. Figure 13 shows examples of the resulting character maps and transition rates. In each case, we simply present the final character map from the 1000 MCMC samples.

3.2 Ebolavirus

Figure 14 supplements Figure 4 from the main text. Shepard plots are provided to indicate the quality of the projections. The distinct VP30 clusters detected by our metric are discernible directly from the distribution of pairwise tree distances (see the Shepard plot: our metric found no tree pairs whose distance was between 7.7 and 10.8). To illustrate the groups of trees (instead of the groups' MCC trees), we have provided their DensiTree [Bouckaert, 2010] plots in Figure 14. These show the same distinct placements of the Sudan clade as those described in the main text.

One difference among the clusters can be interpreted as three different choices of the root for the three major clades. The fact that this uncertainty in the timing of diversification in the ancient history

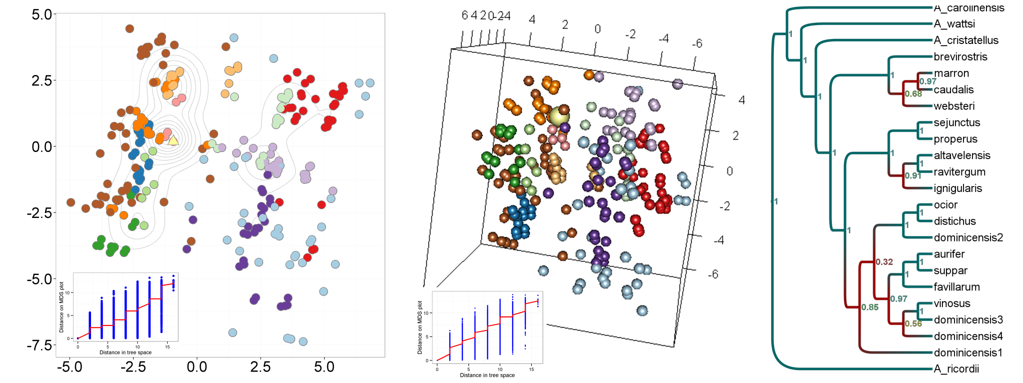

(a) (b) (c) Lilac cluster MCC

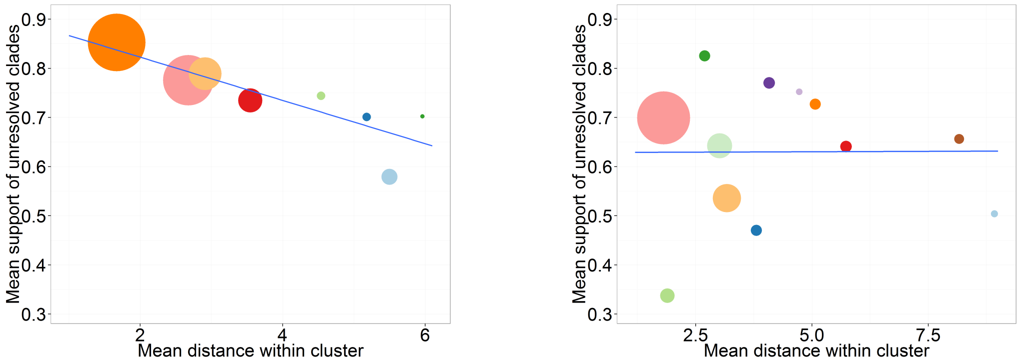

(d) $\lambda = 0$ (e) RF

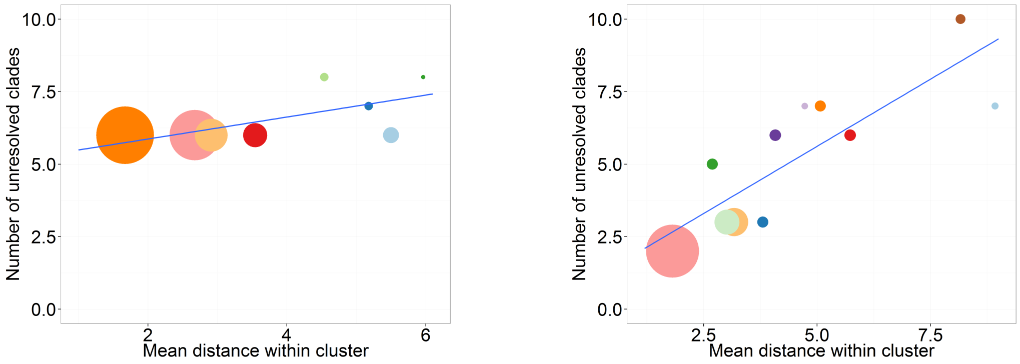

(f) $\lambda = 0$ (g) RF

Figure 11: An analysis of the anoles posterior species trees using the RF metric. MDS plots in 2D (a) and 3D (b) do not show well-separated clusters. (c) MCC tree from the lilac RF cluster, showing multiple areas of uncertainty. (d) and (e) relationship between the spread (mean pairwise distance) within a cluster and the level of certainty in the cluster's MCC tree. The level of certainty is measured by the mean of the cluster's MCC support values that are less than 1. In our metric, the mean support of unresolved clades is highest in tightly-defined clusters (d); this is not the case in the RF metric (e). (f) and (g) compare the spread of a cluster with the number of unresolved clades in its MCC. Our metric shows no strong relationship whereas in the RF metric, clusters with higher spread have more unresolved clades. In (d)-(f), sizes of points correspond to the number of trees in the cluster, and the colours correspond to the MDS clusters for each metric.

occurs in VP30 and not in the other 6 genes is a substantial difference. It is also not the only difference between clusters, as the Sudan 2011 and Reston 1990 placements also varied. The VP30 trees also remain distinctive when lengths are taken into account. Our tree comparison detects the incongruence amongst the posterior trees for VP30, and identifies the three alternative underlying structures, but it does not in and of itself explain why this incongruence has occurred.

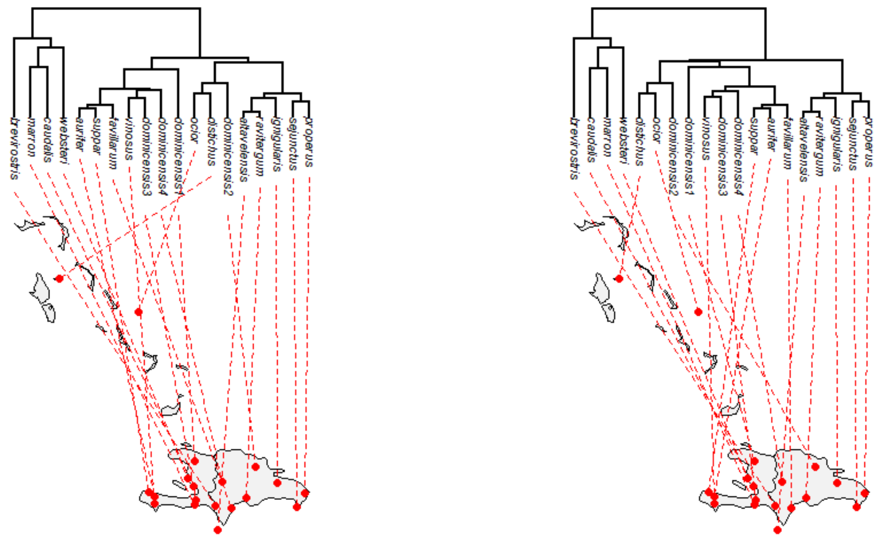

(a) MCC tree from orange cluster, left hand side of MDS plot

(b) Median tree from pale orange cluster, right hand side of MDS plot

Figure 12: Examples of the biogeographic implications of different, likely tree topologies for anole lizards.

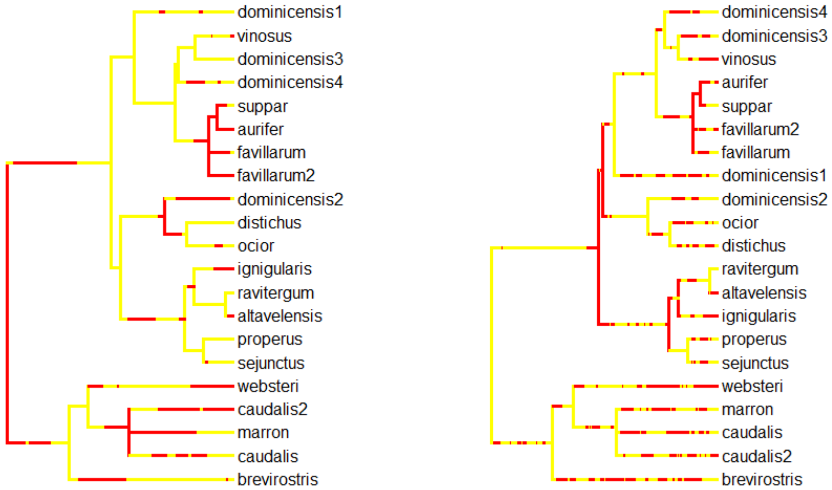

(a) MCC tree from orange cluster. Inferred transition rate: 1.30521

(b) Median tree from pale orange cluster. Inferred transition rate: 1.366596

Figure 13: Stochastic character map estimates of transitions between primarily yellow and primarily dark orange/red dewlap colour in anole lizards.

BEAST estimates of clock rates and the root height differed among the Ebolavirus genes (horizontal scales in the DensiTree plots in Figure 14). When $\lambda = 1$, it is this difference that is primarily detected by the metric and it can also be detected directly from the log files and tree heights. We compared the BHV metric distances to our $\lambda = 1$ (the most comparable alternative as BHV compares rooted trees and captures branch lengths). In both, differences in root heights overwhelmed structural differences in the trees.

3.3 Other datasets

We analysed data from viral and higher organisms to illustrate that mapping the space of phylogenetic trees is a powerful tool in different contexts. Phylogenetic methods face particular challenges in higher organisms. In both viruses and bacteria, genetic variation occurs quickly enough that phylogenetic methods can detect evolution over short time periods using single nucleotide polymorphisms. In most higher organisms diversity accrues more slowly, genomes are larger, and haplotype phasing must be performed

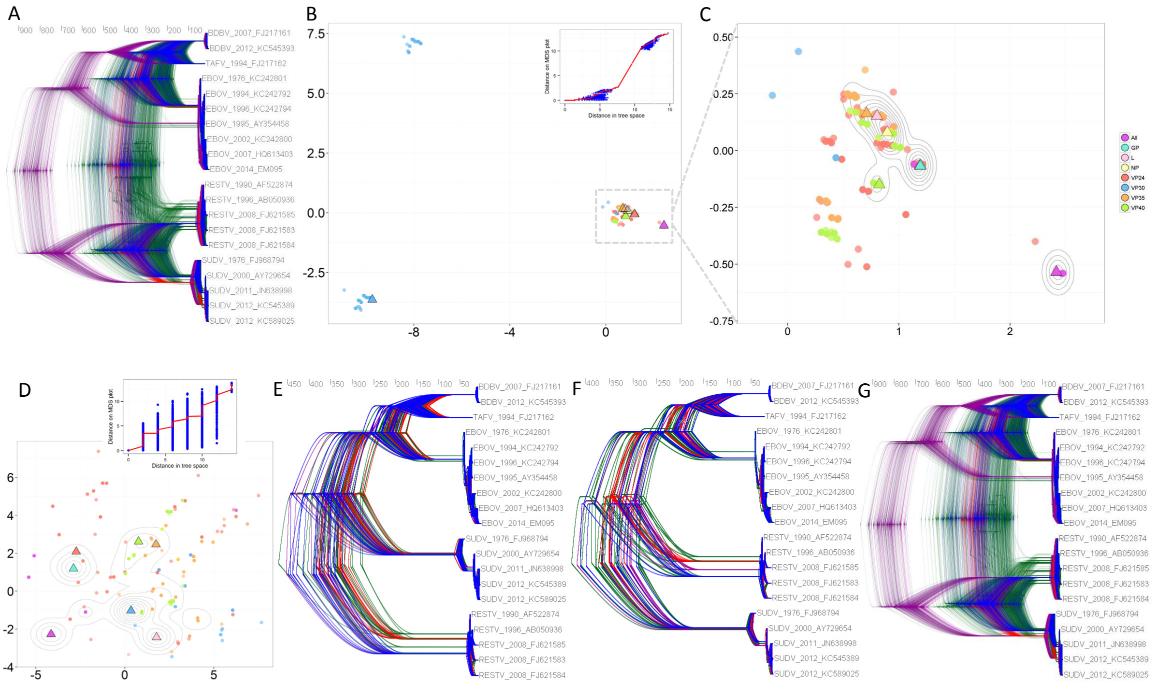

Figure 14: Ebola analysis, parallel to Figure 4 in the main text, with additional Shepard plots. DensiTree [Bouckaert, 2010] images have been used instead of MCC trees to show the resolution of the Sudan clade within each cluster. In a reflection of Figure 4, (A) is a DensiTree image of all 1200 trees from individual genes and from all genes together. (B) is an MDS plot of all trees, coloured by gene. The 'gap' between the majority of trees and the two distinct VP30 clusters is clearly visible in the inset Shepard plot, and is therefore not a spurious result of the projection. (C) gives a closer look at the MDS plot of the majority of trees, as indicated in (B). (D) is the analogous MDS plot to (B), based on RF distances. The collection of trees from each cluster are shown in (E)–(F): (E) is the bottom left VP30 cluster, (F) is the top VP30 cluster and (G) is all the other trees, corresponding to the main cluster.

to determine whether variation at a locus occurs on the same chromosome. Furthermore, while viruses and bacteria do exchange genetic material by routes other than descent (horizontal gene transfer), this is rarer than descent itself. Unlike species' overall ancestry, the ancestry of individual higher organisms is not tree-like because individuals have more than one parent. Estimating species trees from multiple genes sequenced in multiple taxa is a formidable challenge [Nichols, 2001, Degnan and Rosenberg, 2006, Heled and Drummond, 2010, Anderson et al., 2012]. We present here two additional datasets, one in a higher organism (chorus tree frogs) and one in another virus (dengue).

3.3.1 Chorus Frogs

Recently, Barrow et al. used anonymous nuclear loci to estimate a phylogeny for the North America genus of chorus frogs, *Pseudacris* [Barrow et al., 2014a]. As in the case of anole lizards (main text), this genus is a model system with evidence of reproductive character displacement, allopatric divergence, and hybridisation and reinforcement [Fouquette Jr, 1975, Gartside, 1980, Lemmon et al., 2007a, Lemmon et al., 2007b, Lemmon, 2009, Lemmon and Lemmon, 2010]. Data included sequences for 44 individuals from 3 mitochondrial loci and 27 nuclear loci. Full details of the methods and data are available in [Barrow et al., 2014a, Barrow et al., 2014b] respectively. Barrow et al. found that four major clades of frogs were supported consistently but that there was discordance between trees derived from nuclear and mitochondrial data. They interpreted this as a signal of a possible selective sweep, or mitochondrial introgression [Barrow et al., 2014a]. The trees are broadly concordant but there are a number of points of uncertainty in the posterior MCC (Figure 15). We used posterior tree file `species_1367351410414.trees` with the MCC tree `2allele-44taxa-2Kburn.tree`, available in [Barrow et al., 2014b]). We sampled 1000 trees from the posterior, computed tree-tree distances, and visualised the posterior with MDS and k-means clustering.

There are several tightly-defined clusters of distinct trees. Clusters have much higher MCC support values than the MCC for the whole posterior. In particular, clusters differ in whether *triseriata* and *kalmi*

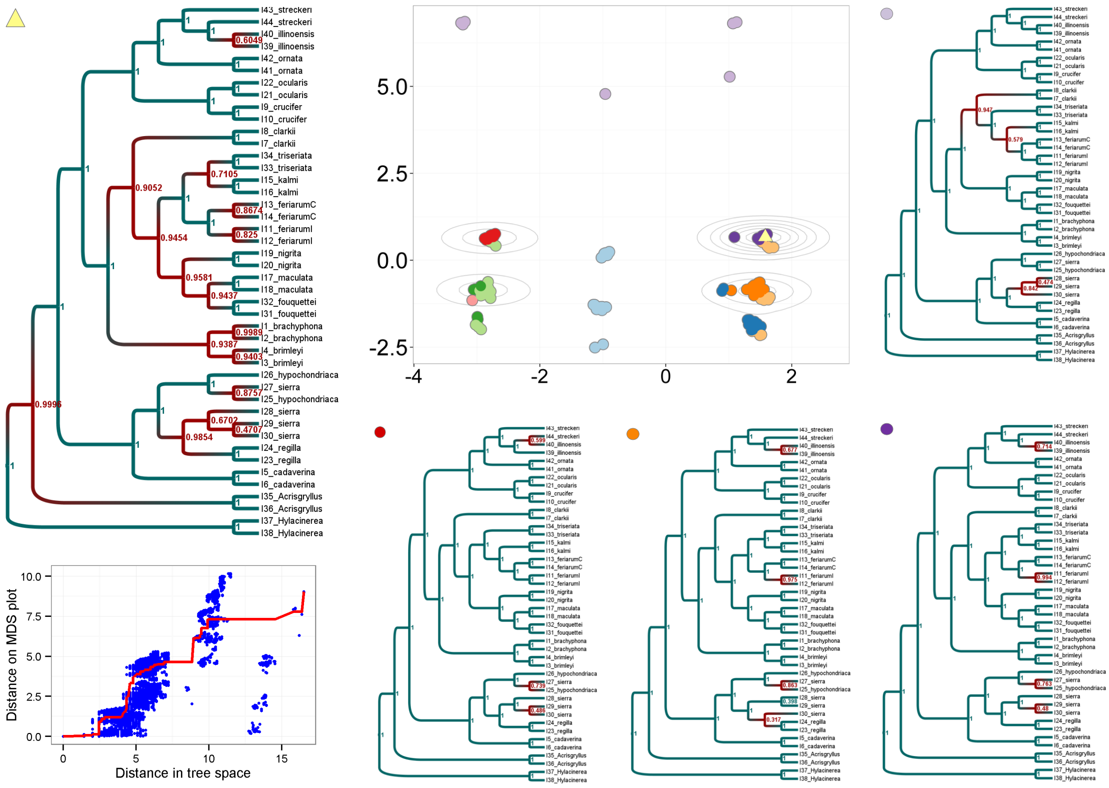

Figure 15: Chorus tree frog analysis showing the correspondence between clusters and distinct topologies. The overall MCC tree is marked by a yellow triangle, and examples of cluster MCC trees are shown, as indicated. Again, the existence of clusters is visible in the Shepard plot in the distribution of distances along the horizontal axis.

are sister clades (as in the posterior MCC, the orange cluster and the purple cluster) or, alternatively, *kalmi*, *ferariumC* and *ferariumI* form a sister clade to *triseriata* (e.g. red cluster, light purple cluster). They also differ in the timing of *clarki*'s divergence from the *brimlei*/*brachyphona* clade, and at several other points. The clusters represent alternative, well-supported patterns in the frogs' evolution.

3.3.2 Dengue

In their paper introducing BEAST [Drummond and Rambaut, 2007], Drummond and Rambaut demonstrated their Bayesian analysis using 17 dengue virus serotype 4 sequences from [Lanciotti et al., 1997] under varying priors for model and clock rate. As a means of comparing posterior tree distributions under different BEAST settings, we ran the `xml` files provided in Drummond and Rambaut's paper [Drummond and Rambaut, 2007] in BEAST v1.8 and compared the resulting trees. Figure 16 shows MDS plots of two of these analyses: Figure 16a is a sample of the posterior under the standard GTR + Γ + I substitution model with uncorrelated lognormal-distributed relaxed molecular clock; Figure 16b is a sample from the posterior under the codon-position specific substitution model GTR + CP, with a strict clock. These analyses demonstrate some of the different signals which can be detected by visualising the metric's tree distances. In particular, they are informative of the extent to which a set of priors constrains the posterior. Distinct clusters are visible in (a), whereas in (b) there are some tight bunches of points (and again, the MCC tree is in the largest cluster) but the posterior is not as clearly separated into distinct clusters. Additionally, trees in (b) are more tightly grouped together overall (the scale of the y-axis) indicating that there is less conflict in the phylogenetic signals in (b). We ran BEAST twice with the settings from (a) using different random starting seeds and found that the space of trees accepted in each run was similar, with the same clusters. It is also encouraging that the MCC tree from the first BEAST run had the same topology as that from the second run, and that this topology again sits in the largest cluster (yellow triangle in Figure 16a).

This simple comparison demonstrates the potential of the method for testing the extent to which priors constrain the posterior, and the stability of an analysis to different starting seeds. We can compare

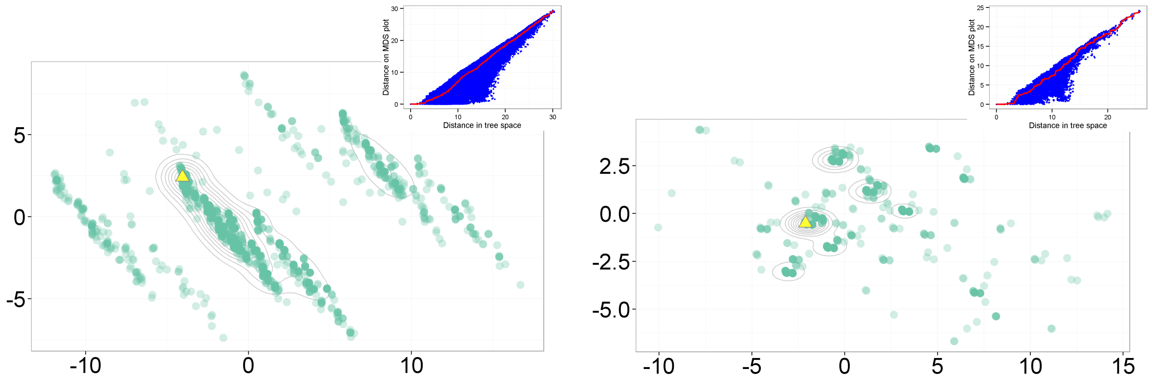

(a) GI relaxed clock, $\lambda = 0$

(b) CP strict clock, $\lambda = 0$

Figure 16: MDS plots of dengue fever trees sampled from posteriors demonstrate differences in the space of trees explored by BEAST under different settings. MCC trees are marked by yellow triangles. (a) GTR + Γ + I substitution model with uncorrelated lognormal-distributed relaxed molecular clock (b) Codon-position specific substitution model GTR + CP, with a strict clock.

different analyses not only by their MCC trees, but also by their ‘spread’ within tree space, the presence of clusters and gaps, multiple representative trees, and so on. Whilst a highly constrained and unimodal posterior cannot *on its own* confirm a good choice of priors, such information can aid in model selection.

References

[Amenta and Klingner, 2002] Amenta, N. and Klingner, J. (2002). Case study: visualizing sets of evolutionary trees. In *IEEE Symposium on Information Visualization, 2002. (InfoVis'02)*, pages 71–74.

[Anderson et al., 2012] Anderson, C. N. K., Liu, L., Pearl, D., and Edwards, S. V. (2012). Tangled trees: the challenge of inferring species trees from coalescent and noncoalescent genes. *Methods in Molecular Biology*, 856:3–28.

[Bacak, 2014] Bacak, M. (2014). Computing medians and means in Hadamard spaces. *SIAM Journal of Optimization*, 24(3):1542–1566.

[Barrow et al., 2014a] Barrow, L. N., Ralicki, H. F., Emme, S. a., and Lemmon, E. M. (2014a). Species tree estimation of North American chorus frogs (Hylidae: Pseudacris) with parallel tagged amplicon sequencing. *Molecular Phylogenetics and Evolution*, 75(1):78–90.

[Barrow et al., 2014b] Barrow, L. N., Ralicki, H. F., Emme, S. A., and Moriarty Lemmon, E. (2014b). Data from Dryad Digital Repository. doi: 10.5061/dryad.23rc0.

[Berglund, 2011] Berglund, D. (2011). *Visualization of Phylogenetic Tree Space*. PhD thesis.

[Billera et al., 2001] Billera, L. J., Holmes, S. P., and Vogtmann, K. (2001). Geometry of the space of phylogenetic trees. *Advances in Applied Mathematics*, 27(4):733–767.

[Bordewich and Semple, 2005] Bordewich, M. and Semple, C. (2005). On the computational complexity of the rooted subtree prune and regraft distance. *Annals of Combinatorics*, 8:409–423.

[Bouckaert, 2010] Bouckaert, R. R. (2010). DensiTree: making sense of sets of phylogenetic trees. *Bioinformatics*, 26(10):1372–1373.

[Boussau et al., 2009] Boussau, B., Guéquen, L., and Gouy, M. (2009). A mixture model and a hidden Markov model to simultaneously detect recombination breakpoints and reconstruct phylogenies. *Evolutionary Bioinformatics*, 25:67–79.

[Brodal et al., 2013] Brodal, G. S., Fagerberg, R., Mailund, T., Pedersen, C. N., and Sand, A. (2013). Efficient algorithms for computing the triplet and quartet distance between trees of arbitrary degree. In *Proceedings of the Twenty-Fourth Annual ACM-SIAM Symposium on Discrete Algorithms*, pages 1814–1832. SIAM.

[Cardona et al., 2013] Cardona, G., Mir, A., Rossello Llompart, F., Rotger, L., and Sanchez, D. (2013). Cophenetic metrics for phylogenetic trees, after Sokal and Rohlf. *BMC Bioinformatics*, 14(1):3.

[Chakerian and Holmes, 2012] Chakerian, J. and Holmes, S. (2012). Computational tools for evaluating phylogenetic and hierarchical clustering trees. *Journal of Computational and Graphical Statistics*, 21(3):581–599.

[Chakerian and Holmes, 2013] Chakerian, J. and Holmes, S. (2013). *distory: Distance Between Phylogenetic Histories*. R package version 1.4.2.

[Chaudhary et al., 2013] Chaudhary, R., Burleigh, J. G., and Fernández-Baca, D. (2013). Inferring species trees from incongruent multi-copy gene trees using the Robinson-Foulds distance. *Algorithms for Molecular Biology*, 8(1):28.

[Choi and Gomez, 2009] Choi, K. and Gomez, S. M. (2009). Comparison of phylogenetic trees through alignment of embedded evolutionary distances. *BMC Bioinformatics*, 10:423.

[Critchlow et al., 1996] Critchlow, D. E., Pearl, D. K., and Qian, C. (1996). The triples distance for rooted bifurcating phylogenetic trees. *Systematic Biology*, 45(3):323–334.

[Croucher et al., 2015] Croucher, N., Page, A., Connor, T., Delaney, A., Keane, J., Bentley, S., Parkhill, J., and Harris, S. (2015). *Rapid phylogenetic analysis of large samples of recombinant bacterial whole genome sequences using Gubbins*. Nucleic acids research 2015;43;3;e15 PUBMED: 25414349; PMC: 4330336; DOI: 10.1093/nar/gku1196.

[de Vienne et al., 2012] de Vienne, D. M., Ollier, S., and Aguileta, G. (2012). Phylo-MCOA: a fast and efficient method to detect outlier genes and species in phylogenomics using multiple co-inertia analysis. *Molecular Biology and Evolution*, 29(6):1587–1598.

[Degnan and Rosenberg, 2006] Degnan, J. H. and Rosenberg, N. A. (2006). Discordance of species trees with their most likely gene trees. *PLoS Genetics*, 2(5):e68.

[Drummond and Rambaut, 2007] Drummond, A. J. and Rambaut, A. (2007). BEAST: Bayesian evolutionary analysis by sampling trees. *BMC Evolutionary Biology*, 7:214.

[Estabrook et al., 1985] Estabrook, G. F., McMorris, F. R., and Meacham, C. A. (1985). Comparison of undirected phylogenetic trees based on subtrees of four evolutionary units. *Systematic Biology*, 34(2):193–200.

[Finden and Gordon, 1985] Finden, C. R. and Gordon, A. D. (1985). Obtaining common pruned trees. *Journal of Classification*, 2(1):255–276.

[Fouquette Jr, 1975] Fouquette Jr, M. J. (1975). Speciation in Chorus Frogs. I. Reproductive Character Displacement in the Pseudacris Nigrita Complex. *Systematic Biology*, 24(1):16–23.

[Gartside, 1980] Gartside, D. F. (1980). Analysis of a Hybrid Zone between Chorus Frogs of the Pseudacris nigrita Complex in the Southern United States. *Copeia*, (1):56–66.

[Geneva et al., 2015] Geneva, A. J., Hilton, J., Noll, S., and Glor, R. E. (2015). Multilocus phylogenetic analyses of Hispaniolan and Bahamian trunk anoles (distichus species group). *Molecular Phylogenetics and Evolution*, 87:105–117.

[Gilks et al., 2006] Gilks, W. R., Nye, T. M. W., and Li, P. (2006). A novel algorithm and web-based tool for comparing two alternative phylogenetic trees. *Bioinformatics*, 22(1):117–119.

[Gori et al., 2016] Gori, K., Suchan, T., Alvarez, N., Goldman, N., and Dessimoz, C. (2016). Clustering genes of common evolutionary history. *Molecular Biology and Evolution*.

[Haldane, 1948] Haldane, J. B. S. (1948). Note on the median of a multivariate distribution. *Biometrika*, 35(3):414–415.

[Harding, 1971] Harding, E. F. (1971). The probabilities of rooted tree-shapes generated by random bifurcation. *Advances in Applied Probability*, 3(1):44–77.

[Haws et al., 2012] Haws, D. C., Huggins, P., O'Neill, E. M., Weisrock, D. W., and Yoshida, R. (2012). A support vector machine based test for incongruence between sets of trees in tree space. *BMC Bioinformatics*, 13:210.

[Heath et al., 2014] Heath, T. A., Huelsenbeck, J. P., and Stadler, T. (2014). The fossilized birth-death process for coherent calibration of divergence-time estimates. *Proceedings of the National Academy of Sciences of the United States of America*, 111(29):E2957–66.

[Hein et al., 1996] Hein, J., Jiang, T., Wang, L., and Zhang, K. (1996). On the complexity of comparing evolutionary trees. *Discrete Applied Mathematics*, 71(1–3):153 – 169.

[Heled and Bouckaert, 2013] Heled, J. and Bouckaert, R. R. (2013). Looking for trees in the forest: summary tree from posterior samples. *BMC Evolutionary Biology*, 13:221.

[Heled and Drummond, 2010] Heled, J. and Drummond, A. J. (2010). Bayesian inference of species trees from multilocus data. *Molecular Biology and Evolution*, 27(3):570–580.

[Hillis et al., 2005] Hillis, D. M., Heath, T. A., and St John, K. (2005). Analysis and visualization of tree space. *Systematic Biology*, 54(3):471–482.

[Holland et al., 2007] Holland, B., Conner, G., Huber, K., and Moulton, V. (2007). Imputing supertrees and supernetworks from quartets. *Systematic Biology*, 56(1):57–67.

[Holmes, 2006] Holmes, S. (2006). Visualising data. In Lyons, L. and Ünel, M. K., editors, *Statistical Problems in Particle Physics, Astrophysics and Cosmology, Proceedings of PHYSTAT05*, pages 197–208. Imperial College Press.

[Jombart and Dray, 2010] Jombart, T. and Dray, S. (2010). adephylo: exploratory analyses for the phylogenetic comparative method. *Bioinformatics*, 26:1907–1909.

[Jombart et al., 2015] Jombart, T., Kendall, M., Almagro-Garcia, J., and Colijn, C. (2015). *treescape: Statistical Exploration of Landscapes of Phylogenetic Trees*. R package version 1.8.15.

[Kishino and Hasegawa, 1989] Kishino, H. and Hasegawa, M. (1989). Evaluation of the maximum likelihood estimate of the evolutionary tree topologies from DNA sequence data, and the branching order in hominoidea. *Journal of Molecular Evolution*, 29(2):170–179.

[Koonin et al., 2011] Koonin, E. V., Puigbò, P., and Wolf, Y. I. (2011). Comparison of phylogenetic trees and search for a central trend in the "forest of life". *Journal of Computational Biology*, 18(7):917–924.

[Kuhner and Felsenstein, 1994] Kuhner, M. K. and Felsenstein, J. (1994). A simulation comparison of phylogeny algorithms under equal and unequal evolutionary rates. *Molecular Biology and Evolution*, 11(3):459–468.

[Kuhner and Yamato, 2014] Kuhner, M. K. and Yamato, J. (2014). Practical performance of tree comparison metrics. *Systematic Biology*, 64(2):205–214.

[Lanciotti et al., 1997] Lanciotti, R. S., Gubler, D. J., and Trent, D. W. (1997). Molecular evolution and phylogeny of dengue-4 viruses. *Journal of General Virology*, 78(9):2279–2286.

[Leigh et al., 2011] Leigh, J. W., Schliep, K., Lopez, P., and Bapteste, E. (2011). Let them fall where they may: congruence analysis in massive phylogenetically messy data sets. *Molecular Biology and Evolution*, 28(10):2773–2785.

[Leigh et al., 2008] Leigh, J. W., Susko, E., Baumgartner, M., and Roger, A. J. (2008). Testing congruence in phylogenomic analysis. *Systematic Biology*, 57(1):104–115.

[Lemmon, 2009] Lemmon, E. M. (2009). Diversification of conspecific signals in sympatry: Geographic overlap drives multidimensional reproductive character displacement in frogs. *Evolution*, 63(5):1155–1170.

[Lemmon and Lemmon, 2010] Lemmon, E. M. and Lemmon, A. R. (2010). Reinforcement in chorus frogs: Lifetime fitness estimates including intrinsic natural selection and sexual selection against hybrids. *Evolution*, 64(6):1748–1761.

[Lemmon et al., 2007a] Lemmon, E. M., Lemmon, A. R., and Cannatella, D. C. (2007a). Geological and climatic forces driving speciation in the continentally distributed trilling chorus frogs (Pseudacris). *Evolution*, 61(9):2086–2103.

[Lemmon et al., 2007b] Lemmon, E. M., Lemmon, A. R., Collins, J. T., Lee-Yaw, J. a., and Cannatella, D. C. (2007b). Phylogeny-based delimitation of species boundaries and contact zones in the trilling chorus frogs (Pseudacris). *Molecular Phylogenetics and Evolution*, 44(3):1068–1082.

[Lewitus and Morlon, 2015] Lewitus, E. and Morlon, H. (2015). Characterizing and comparing phylogenies from their Laplacian spectrum.

[Liebscher, 2015] Liebscher, V. (2015). Gromov meets phylogenetics — new animals for the zoo of biocomputable metrics on tree space. *arXiv preprint arXiv:1504.05795v1*.

[Maddison, 1991] Maddison, D. R. (1991). The discovery and importance of multiple islands of most-parsimonious trees. *Systematic Zoology*, 40(3):315–328.

[Nichols, 2001] Nichols, R. (2001). Gene trees and species trees are not the same. *Trends in Ecology and Evolution*, 16(7):358–364.

[Nye, 2008] Nye, T. M. W. (2008). Trees of trees: an approach to comparing multiple alternative phylogenies. *Systematic Biology*, 57(5):785–794.

[Pond et al., 2006] Pond, S. L. K., Posada, D., Gravenor, M. B., Woelk, C. H., and Frost, S. D. (2006). GARD: a genetic algorithm for recombination detection. *Bioinformatics*, 22:3096–8.

[Poon et al., 2013] Poon, A. F. Y., Walker, L. W., Murray, H., McCloskey, R. M., Harrigan, P. R., and Liang, R. H. (2013). Mapping the shapes of phylogenetic trees from human and zoonotic RNA viruses. *PloS One*, 8(11):e78122.

[Revell, 2012] Revell, L. J. (2012). phytools: An r package for phylogenetic comparative biology (and other things). *Methods in Ecology and Evolution*, 3:217–223.

[Robinson and Foulds, 1979] Robinson, D. F. and Foulds, L. R. (1979). Comparison of weighted labelled trees. *Lecture Notes in Mathematics*, 748:119–126.

[Robinson and Foulds, 1981] Robinson, D. F. and Foulds, L. R. (1981). Comparison of phylogenetic trees. *Mathematical Biosciences*, 53(1-2):131–147.

[Salichos et al., 2014] Salichos, L., Stamatakis, A., and Rokas, A. (2014). Novel information theory-based measures for quantifying incongruence among phylogenetic trees. *Molecular Biology and Evolution*, 31(5):1261–1271.

[Salter and Pearl, 2001] Salter, L. A. and Pearl, D. K. (2001). Stochastic search strategy for estimation of maximum likelihood phylogenetic trees. *Systematic Biology*, 50(1):7–17.

[Sanderson et al., 2011] Sanderson, M. J., McMahon, M. M., and Steel, M. (2011). Terraces in phylogenetic tree space. *Science*, 333(6041):448–450.

[Schliep, 2011] Schliep, K. (2011). phangorn: phylogenetic analysis in r. *Bioinformatics*, 27(4):592–593.

[Shepard et al., 1972] Shepard, R. N., Romney, A. K., and Nerlove, S. B. (1972). Multidimensional Scaling: Theory and applications in the behavioural sciences: I. Theory. Seminar press.

[Shimodaira, 2002] Shimodaira, H. (2002). An approximately unbiased test of phylogenetic tree selection. *Systematic Biology*, 51(3):492–508.

[Shimodaira and Hasegawa, 1999] Shimodaira, H. and Hasegawa, M. (1999). Multiple comparisons of log-likelihoods with applications to phylogenetic inference. *Molecular Biology and Evolution*, 16:1114–1116.

[Sokal and Rohlf, 1962] Sokal, R. R. and Rohlf, F. J. (1962). The comparison of dendrograms by objective methods. *Taxon*, 11:33–40.

[Steel and Penny, 1993] Steel, M. A. and Penny, D. (1993). Distributions of tree comparison metrics - some new results. *Systematic Biology*, 42(2):126–141.

[Stenetorp et al., 2012] Stenetorp, P., Pyysalo, S., Topić, G., Ohta, T., Ananiadou, S., and Tsujii, J. (2012). Brat: A web-based tool for nlp-assisted text annotation. In *Proceedings of the Demonstrations at the 13th Conference of the European Chapter of the Association for Computational Linguistics*, EACL '12, pages 102–107, Stroudsburg, PA, USA. Association for Computational Linguistics.

[Sullivan et al., 1996] Sullivan, J., Holsinger, K. E., and Simon, C. (1996). The effect of topology on estimates of among-site rate variation. *Journal of Molecular Evolution*, 42(2):308–312.

[Weyenberg et al., 2014] Weyenberg, G., Huggins, P. M., Schardl, C. L., Howe, D. K., and Yoshida, R. (2014). kdetrees: Non-parametric estimation of phylogenetic tree distributions. *Bioinformatics*, 30(16):2280–2287.

[Whidden and Matsen IV, 2015] Whidden, C. and Matsen IV, F. A. (2015). Calculating the unrooted subtree prune-and-regraft distance. *arXiv preprint arXiv:1511.07529*.

[Williams and Clifford, 1971] Williams, W. T. and Clifford, H. T. (1971). On the comparison of two classifications of the same set of elements. *Taxon*, 20(4):519–522.
